# Predictive validity of consensus-based MRI definition of osteoarthritis plus radiographic osteoarthritis for the progression of knee osteoarthritis: A longitudinal cohort study

**DOI:** 10.1016/j.ocarto.2025.100582

**Published:** 2025-02-15

**Authors:** Xing Xing, Yining Wang, Jianan Zhu, Ziyuan Shen, Flavia Cicuttini, Graeme Jones, Dawn Aitken, Guoqi Cai

**Affiliations:** a Department of Epidemiology and Biostatistics, School of Public Health, Anhui Medical University, Hefei, Anhui, China; b Department of Biostatistics, Johns Hopkins Bloomberg School of Public Health, Baltimore, MD, USA; c Menzies Institute for Medical Research, University of Tasmania, Hobart, Tasmania, Australia; d Department of Biostatistics, School of Public Health, New York University, New York, NY, USA; e School of Public Health and Preventive Medicine, Monash University, Melbourne, Australia

**Keywords:** Cartilage volume, Knee osteoarthritis, Machine learning, MRI, Pain, Radiograph, Total knee replacement

## Abstract

**Objective:**

Our previous study showed that magnetic resonance imaging (MRI)-defined tibiofemoral osteoarthritis (MRI-OA), based on a Delphi approach, in combination with radiographic OA (ROA) had a strong predictive validity for the progression of knee OA. This study aimed to compare whether the combination using traditional prediction models was superior to the Light Gradient Boosting Machine (LightGBM) models.

**Methods:**

Data were from the Tasmanian Older Adult Cohort. A radiograph and 1.5T MRI of the right knee was performed. Tibial cartilage volume was measured at baseline, 2.6 and 10.7 years. Knee pain and function were assessed at baseline, 2.6, 5.1, and 10.7 years. Right-sided total knee replacement (TKR) were assessed over 13.5 years. The area under the curve (AUC) was applied to compare the predictive validity of logistic regression with the LightGBM algorithm. For significant imbalanced outcomes, the area under the precision-recall curve (AUC-PR) was used.

**Results:**

574 participants (mean 62 years, 49 ​% female) were included. Overall, the LightGBM showed a clinically acceptable predictive performance for all outcomes but TKR. For knee pain and function, LightGBM showed better predictive performance than logistic regression model (AUC: 0.731–0.912 vs 0.627–0.755). Similar results were found for tibial cartilage loss over 2.6 (AUC: 0.845 vs 0.701, p ​< ​0.001) and 10.7 years (AUC: 0.845 vs 0.753, p ​= ​0.016). For TKR, which exhibited significant class imbalance, both algorithms performed poorly (AUC-PR: 0.647 vs 0.610).

**Conclusion:**

Compared to logistic regression combining MRI-OA, ROA, and common covariates, LightGBM offers valuable insights that can inform early risk identification and targeted prevention strategies.

## 1 Introduction

Knee osteoarthritis (OA) is a common joint disease characterized by knee pain, disability, disability, and the deterioration of articular cartilage. Early diagnosis of OA is essential for initiating timely interventions, thereby slowing the progression of the disease trajectory [1]. The morphological assessment of OA highly relies on radiography, which has limited sensitivity and does not manifest in the early stage [2]. Magnetic resonance imaging (MRI) is regarded as a promising tool for screening early OA as it enables direct visualization of many soft tissue changes proceeding radiographic abnormalities. However, it is difficult to incorporate complex MRI-detected structural changes for defining early OA and predicting disease progression.

In 2011, the Osteoarthritis Research Society International (OARSI) OA Imaging Working Group developed an MRI definition of structural OA (MRI-OA), using a Delphi approach [3]. This is an important attempt to integrate MRI features for defining early OA. A recent cross-sectional study compared the MRI-OA definition with different combinations of MRI features including the presence of either bone marrow lesions (BMLs) or synovitis in addition to cartilage damage (a score ≥2) and osteophytes using the Whole-Organ MRI Score system [4]. They found that compared to simple combinations of MRI features, the MRI-OA showed similar performance in identifying prevalent radiographic OA (ROA) and symptomatic OA but was more complex to apply [4]. In our previous study, we compared the predictive validity of the MRI-OA with traditional ROA for the progression of OA [5]. The results indicated that MRI-OA alone did not outperform ROA in predicting either structural or symptomatic OA progression. However, the combination of MRI-OA and ROA demonstrated significantly enhanced predictive validity [5]. These findings indicate that MRI features can provide valuable insights for defining OA and predicting its progression over time; however, only a limited number of MRI features may contribute to this process, making data-driven approaches advantageous in identifying and incorporating these specific features.

Machine learning (ML) is a powerful tool to decipher complex diseases such as OA [6,7]. While ML algorithms have been applied to develop predictive models for OA progression [8,9], few studies have combined MRI features with demographic and clinical factors at the same time [10]. Moreover, existing studies were mostly conducted in populations from Europe and the United States and had a relatively short follow-up period [[6], [7], [8]]. Therefore, using data from a 10.7-year longitudinal cohort in Australia, our study aimed to compare the predictive validity of traditional vs ML models for predicting the progression of knee OA over time.

## 2 Methods

### 2.1 Data source

This study is reported according to the Transparent reporting of a multivariable prediction model for individual prognosis or diagnosis (TRIPOD) statement [11]. The data souce of this study was the Tasmania Older Adult Cohort (TASOAC), a longitudinal, prospective observational study involving community-dwelling older adults. Between March 2002 and September 2004, 1099 individuals out of 1904 older adults were randomly chosen from the electoral roll in Southern Tasmania (population 229,000) and enrolled in this cohort, resulting in a response rate of 57 ​%. Subsequently, three follow-up assessments were carried out at intervals of 2.6, 5.1, and 10.7 years. In the present study, 574 participants who possessed adequate data for the assessment of MRI-OA and ROA at baseline were incorporated (see flowchart in Supplementary Fig. 1). Ethics approval was obtained from the Southern Tasmanian Health and Medical Human Research Ethics Committee, and written informed consent was provided by all participants.

### 2.2 Predictors

All predictors were selected based on their potential association with the progression of OA, according to previous literature in combination with our research experience. MRI scans of the right knee were conducted at baseline utilizing a 1.5T whole-body magnetic resonance unit (Picker, Cleveland, Ohio, USA). However, at 2.6 years, this machine was utilized for only 60 ​% (345 out of 574) of participants due to its decommissioning halfway through the follow-up period. At the 10.7-year follow-up, a different 1.5T whole-body MRI unit (Siemens, Espree, Pennsylvania, USA) was employed for 66 ​% (377 out of 574) of participants, primarily due to loss to follow-up or other reasons. Both MRI units utilized a commercial transmit-receive extremity coil. Detailed descriptions of sagittal image sequences and technical parameters were provided previously [5]. The measured MRI features included osteophytes, cartilage lesions, meniscal lesions, effusion-synovitis, and subchondral BML (details see Supplementary text). According to the Delphi method [3], the definition of MRI-OA is: the presence of well-defined osteophytes and full-thickness cartilage loss, or either of these features in combination with at least two of the following: a) Subchondral bone marrow lesions (BML) or cysts not associated with meniscal or ligamentous attachments; b) Meniscal subluxation, maceration, or degenerative (horizontal) tear; c) Partial-thickness cartilage loss.

Radiographic evaluation of the right knee was conducted in a standing anteroposterior semi-flexed view at baseline. Osteophytes and joint space narrowing (JSN) in the medial and lateral compartments were examined and scored on a scale of 0–3 according to the OARSI atlas [12]. The intra-observer reliability, as assessed by the Intraclass Correlation Coefficient (ICC), ranged from 0.65 to 0.85 [13]. ROA was deemed present if any of the following criteria were fulfilled in either the medial or lateral compartments: a) the JSN grade ≥ 2; b) the summary grades of osteophytes ≥ 2; or c) grade one JSN was accompanied by grade one osteophytes [14].

Demographic, anthropometric, and lifestyle-related data included age, sex and body mass index (BMI, kg/m^2^), employment status, history of knee surgery and injury, knee symptoms, smoking status (former, current, or never), physical activity (steps/day), socioeconomic status, pain-relief medication, and comorbidity at baseline (details see Supplementary Text and Supplementary Table 1).

### 2.3 Outcome measures

The following outcomes were used as measures of the onset and progression of OA: 1) incident knee pain/disability at 2.6, 5.1, and 10.7 years (in participants without knee pain/disability at baseline); 2) progression of knee pain/disability at 2.6, 5.1 and 10.7 years (in participants with any knee pain/disability at baseline); 3) risk of total knee replacement (TKR) over 13.5 years; and 4) risk of 1 ​%/year loss in tibial cartilage volume over 2.6 and 10.7 years.

Knee pain and disability were measured using the Western Ontario and McMaster Universities Osteoarthritis Index (WOMAC) pain and function subscales at baseline, 2.6, 5.1, and 10.7 years. The WOMAC consists of five items for knee pain and 17 for function, each scored from 0 to 9. This leads to a subscale score range of 0–45 for knee pain and 0–153 for disability, where higher scores denote increased severity. Participants were categorized based on the presence or absence of knee pain (WOMAC pain score ≥1) at baseline. The initiation of knee pain was delineated by the presence of knee pain (WOMAC pain score ≥1) during follow-up among those who were pain-free at baseline. The minimal clinically important difference for this population was calculated to be 0.9, which corresponds to a 12 ​% change of the mean level of baseline pain [15,16]. Hence, we characterized the advancement of knee pain as any elevation in WOMAC pain of ≥1. This approach was likewise utilized to define the absence, onset, and progression of disability through the WOMAC function subscale, where progression of function was designated by any increase in WOMAC function score of ≥3.

Data on TKR were abstained from the Australian Orthopaedic Association National Joint Replacement Registry (AOANJRR) from March 1, 2002 to September 21, 2016 [17]. In this study, all right-sided TKR procedures conducted for OA were considered.

Cartilage volume (mm^3^) of both the tibial and femoral regions was quantified from T1-weighted MRI scans at baseline and 2.6 years, with assessments conducted in a blinded manner to both chronological order and participants' identification [18]. Moreover, a comprehensive new paired evaluation of tibial cartilage volume was undertaken in 377 (65.7 ​%) participants possessing MRI data at both baseline and 10.7 years, with the chronological order disclosed to the reader. The volumes of individual cartilage plates, including those of the medial and lateral compartments, were delineated from the total volume by manually tracing disarticulation contours around the cartilage boundaries on a section-by-section basis. Subsequently, The data underwent resampling using bilinear and cubic interpolation methods, resulting in continuous sections with an area of 312 ​× ​312 ​mm and a thickness of 1.5 ​mm for the final 3-dimensional rendering. The coefficient of variation varied within the range of 2.1 ​%–2.2 ​% [18]. Femoral cartilage volume was not involved in our study due to no measurement in the paired reading (baseline and 10.7 years) but a previous study has demonstrated that longitudinal changes in femoral and tibial cartilage volume exhibit a significant correlation [17]. In this study, only tibial cartilage volume was used as the outcome measure, and femoral cartilage volume at baseline was used as a predictor in the ML models. For both unpaired (baseline and 2.6 years) and paired (baseline and 10.7 years) measurements, the annual percentage change in tibial cartilage volume (%/year) was computed as follows: 100 ​× ​[(follow-up volume - baseline volume)/baseline volume]/time between two scans in years. Though a precise minimal clinically important difference in cartilage loss hasn't been defined, it is noteworthy that cartilage loss has been linked to a heightened risk of knee replacement. Remarkably, a 1 ​% increase in cartilage loss per year correlates with a 20 ​% higher risk of knee replacement surgery over a 4-year period [19]. Therefore, we defined progressive and non-progressive OA as ​≥ ​and <1 ​%/year loss in cartilage volume, respectively.

### 2.4 Statistical analysis

Baseline characteristics of participants were described using mean ​± ​standard deviation (SD) for continuous variables and n (%) for categorical variables, stratified by the presence of MRI-OA.

For the onset and progression of knee pain and functional disability, the risk of ≥1 ​%/year tibial cartilage loss, and the risk of TKR, we built logistic regression models to evaluate the predictive validity of the combination of MRI-OA, ROA features (JSN and osteophytes grades, 0–3), and common covariates, which were identified according to previous literature and a directed acyclic graph (DAG) (Supplementary Fig. 2) [5,[20], [21], [22]].

We built ML models using the Light Gradient Boosting Machine (LightGBM) algorithm with Decision Trees as Base Learners [23]. LightGBM employs weak classifiers, typically decision trees, in an iterative training process to achieve an optimal model. This approach offers the advantages of producing effective training results while minimizing the likelihood of overfitting. LightGBM performs embedded feature selection as part of its training process by assigning importance scores to features through gradient-based optimization, and we did not perform a separate feature selection such as lasso regression. Features with minimal predictive power naturally receive lower importance or are effectively ignored, which reduces the need for an explicit feature selection step. For the LightGBM models, predictor features included individual MRI and radiographic features (JSN and osteophytes grades), demographics, anthropometrics, and lifestyle-related data (Supplementary Table 2). To evaluate the predictive validity of the tested prediction models, the dataset was partitioned into 70 ​% training and 30 ​% testing subsets. RandomSearch and 5-fold cross-validation were utilized to identify the optimal parameters and hyperparameters for each model. The results of the 5-fold cross-validation during hyperparameter tuning are presented in Supplementary Table 3. After determining the optimal hyperparameters, the model was retrained on the complete training subset (70 ​% of the data) using the selected optimal hyperparameters. The final evaluation was then performed on the testing subset (30 ​% of the data) to assess the model's predictive performance. Several adjustable parameters and hyperparameters were fine-tuned to optimize performance and accommodate data imbalance (Supplementary Table 4). For imbalanced dataset, AUC-PR was used as the primary metric for hyperparameter tuning to account for class imbalance. To further manage the imbalance of the outcomes, we adjusted scale_pos_weight parameter, which modifies the loss function to emphasize the minority class. This adjustment helps the model learn more effectively from the limited positive cases without overfitting to the majority class.

**Fig. 1:**
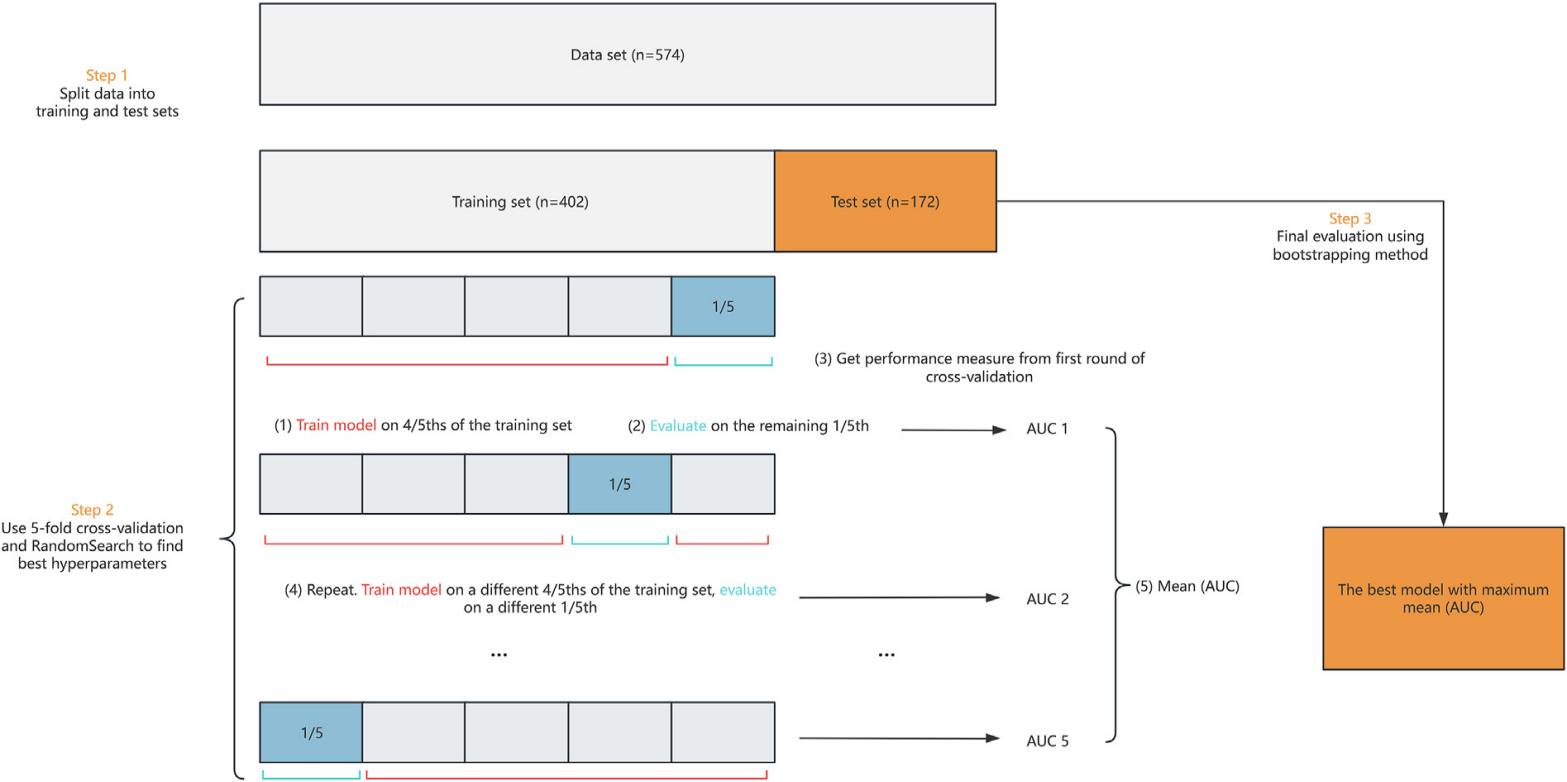
The flowchart for conducting the Machine Learning Model.

For model testing, we used the bias-corrected and accelerated (BCa) bootstrap method, which involved 1000 bootstrap resamples. We imputed missing values using the MissForest method [24] and incorporated them into the 5-fold cross-validation. Besides, we used one-hot encoding for categorical variables. All preprocessing decisions (scaling, feature selection, missing value imputation) were taken based only on training data. The flowchart for conducting the ML is shown in Fig. 1.

For LightGBM and logistic regression models that combined MRI-OA, ROA, and common covariates, the area under the curve (AUC), positive predictive value (PPV), negative predictive value (NPV), sensitivity and specificity, and accuracy were calculated on the holdout test sets to evaluate model performance. For outcomes with significant imbalance, we applied the area under the precision-recall curve (AUC-PR) as the main results. A model with an AUC >0.7 was considered clinically acceptable [25]. F1 scores, derived from a harmonic balance between precision and recall (sensitivity), were computed alongside other top-performing metrics to gauge positive predictive power. The identification of key predictors within the leading model was accomplished through variable importance evaluation functions inherent to the employed machine learning models. Differences in AUC between logistic regression models and ML models were assessed using DeLong's test [26], where p-values were adjusted for using Bonferroni's method to account for multiple tests. A significance level of p-value of <0.05 was considered statistically significant. SHAP (SHapley Additive exPlanations) is a game-theoretic approach that provides explanations for model predictions, offering detailed insights into how features contribute to individual predictions. We conducted the SHAP to generate more information of how these models take decisions. All the analysis was conducted in Python 3.10.7 (package scikit-learn 1.2.2. and lightgbm 3.3.5) and R 4.2.3.

## 3 Results

### 3.1 Participants characteristics

Out of the 574 participants included, 159 (27.7 ​%) had MRI-OA. The characteristics of participants are shown in Table 1, split by MRI-OA (yes/no). Overall, 291 (50.6 ​%) and 324 (56.4 ​%) participants had knee pain and disability at baseline, respectively, and 175 (30.4 ​%) and 240 (41.8 ​%) experienced at least 1 ​%/year loss in tibial cartilage volume over 2.6 and 10.7 years, respectively. The summary information of missing data in predictors is presented in Supplementary Table 2. During the follow-up, 19.1%–19.9 ​% and 20.0%–23.5 ​% of participants had incident knee pain and functional disability, respectively, in those without knee pain and functional disability at baseline (Supplementary Table 5). Among those who had knee pain or functional disability at baseline, 25.2%–26.2 ​% and 20.4%–26.5 ​% had worse knee pain and functional disability, respectively. Eighteen (3.14 ​%) participants underwent right-sided TKR over a mean follow-up of 13.5 years (Supplementary Table 5).

**Table 1:** Baseline characteristics of study participants.

	Total	MRI-OA	
	N ​= ​574	No (n ​= ​415)	Yes (n ​= ​159)
Age, year, mean (SD)	62.47 (7.3)	61.74 (7.0)	64.4 (7.7)
Body mass index, kg/m^2^, mean (SD)	28.6 (4.8)	27.1 (4.0)	29.5 (5.1)
Gender, n (%)			
Male	292 (50.9 ​%)	197 (67.5 ​%)	95 (32.5 ​%)
Female	282 (49.1 ​%)	218 (77.3 ​%)	64 (22.7 ​%)
WOMAC pain, mean (SD) (range 0–45)	3.3 (6.0)	2.69 (5.5)	4.89 (7.0)
WOMAC function, mean (SD) (range 0–153)	10.74 (21.0)	8.14 (18.5)	17.53 (25.4)
Physical activity (steps/day), mean (SD)	9000.56 (3320.6)	9104.63 (3131.0)	8728.92 (3768.1)
Tibiofemoral cartilage volume (mm^3^), mean (SD)	13610.94 (3253.7)	13454.66 (3350.8)	14018.82 (2956.5)
Tibial cartilage volume (mm^3^), mean (SD)	3552.6 (884.1)	3520.17 (907.0)	3637.25 (817.9)
Joint space narrowing			
Grade 0	223 (38.9 ​%)	184 (82.5 ​%)	39 (17.5 ​%)
Grade 1	242 (42.2 ​%)	193 (79.8 ​%)	49 (20.2 ​%)
Grade 2	78 (13.6 ​%)	33 (42.3 ​%)	45 (57.7 ​%)
Grade 3	31 (5.4 ​%)	5 (16.1 ​%)	26 (83.9 ​%)
Osteophytes			
Grade 0	505 (88 ​%)	397 (78.6 ​%)	108 (21.4 ​%)
Grade 1	38 (6.6 ​%)	15 (39.5 ​%)	23 (60.5 ​%)
Grade 2	22 (3.8 ​%)	3 (13.6 ​%)	19 (86.4 ​%)
Grade 3	9 (1.6 ​%)	0 (0 ​%)	9 (100 ​%)
Employed	224 (39 ​%)	148 (66.1 ​%)	76 (33.9 ​%)
Any history of knee surgery, n (%)	69 (12 ​%)	37 (53.6 ​%)	32 (46.4 ​%)
Any history of knee injury, n (%)	67 (11.7 ​%)	39 (58.2 ​%)	28 (41.8 ​%)
Consumed any pain-relief medication, n (%)	314 (54.7 ​%)	225 (71.7 ​%)	89 (28.3 ​%)
Any comorbidity, n (%)	185 (32.2 ​%)	129 (69.7 ​%)	56 (30.3 ​%)

SD, standard deviation; MRI-OA, magnetic resonance imaging-defined tibiofemoral osteoarthritis; WOMAC, Western Ontario and McMaster Universities Osteoarthritis.

### 3.2 Predictive validity for the onset and progression of knee symptoms

**Fig. 2:**
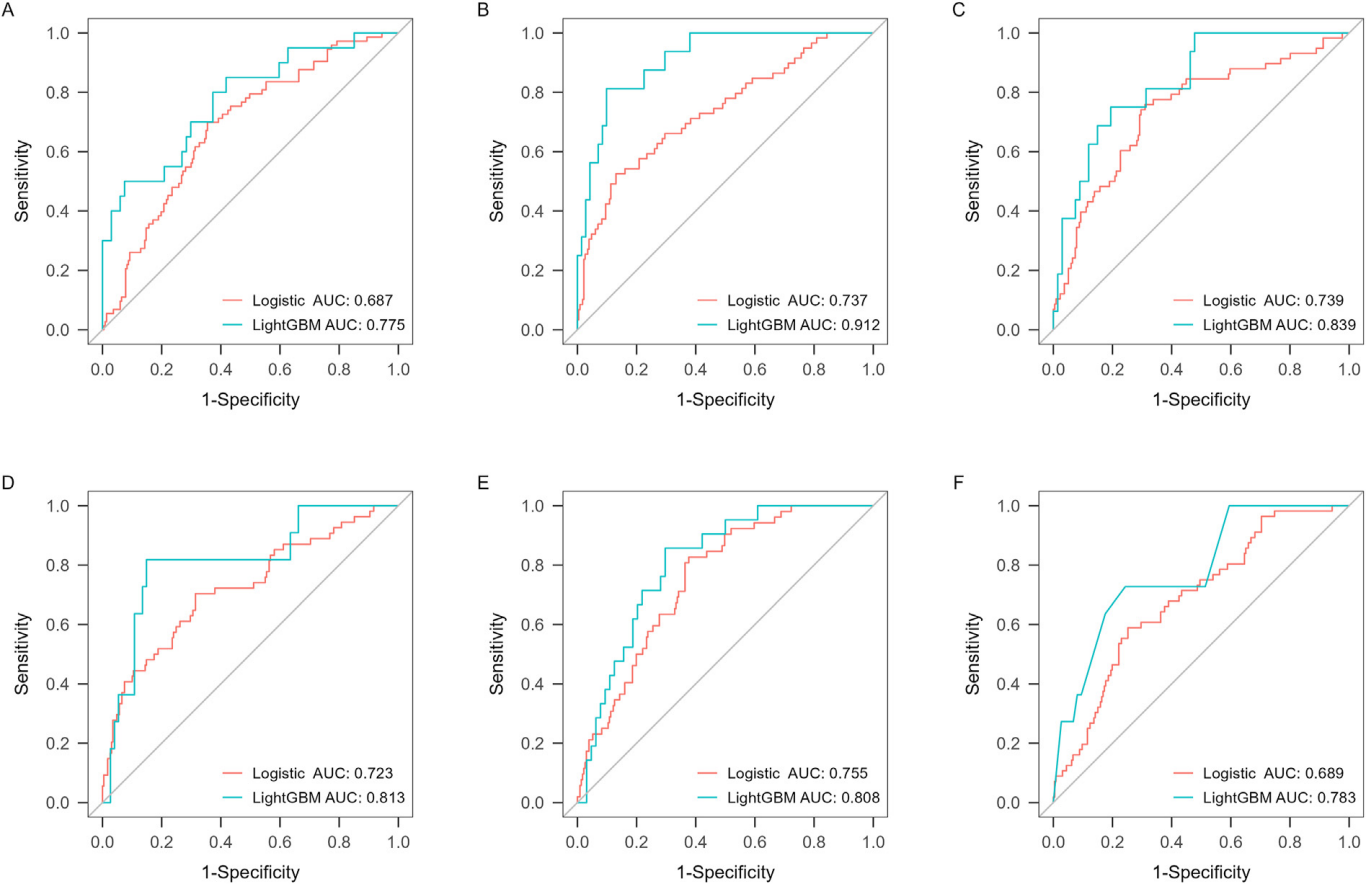
ROC curves for LightGBM and logistic regression models for prediction of progression of symptoms in pain at 2.6 (A), 5.1 (B) and 10.7 (C) years and onset of symptoms in pain at 2.6 (D), 5.1 (E) and 10.7 (F) years.

**Fig. 3:**
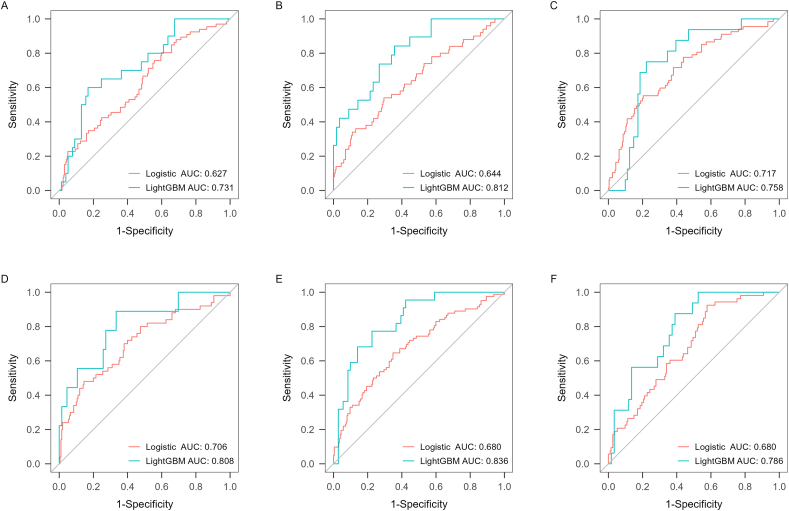
Predictive validity of LightGBM and logistic regression models for the onset (A–C) and progression (D–F) of functional disability at 2.6, 5.1, and 10.7 years, respectively.

The AUCs of LightGBM and logistic regression models for predicting the onset and progression of knee pain and functional disability over 2.6, 5.1, and 10.7 years were 0.731–0.912 and 0.627–0.755, respectively (Fig. 2, Fig. 3 and Table 2). Overall, the predictive validity of the LightGBM model was stronger than logistic regression models for all knee symptom outcome measures, although statistical significance was not reached for most comparisons of AUCs after the Bonferroni adjustment (Table 2).

**Table 2:** Discrimination performance of machine learning models (LightGBM) and logistic regression models (MRI-OA, ROA, and common covariates) for the progression of knee osteoarthritis.

Outcomes	Models	AUC (95 ​% CI)		Sensitivity	Specificity	PPV	NPV	F1	Accuracy	P-value
**Incidence of TKR over 13.5 years**	LightGBM	0.647^a^	–	0.875	0.830	0.200	0.993	0.326	0.832	–
	Logistic regression	0.610^a^	–	0.944	0.888	0.215	0.998	0.350	0.890	
**Cartilage volume loss (≥ or < 1 ​%/year)**										
Tibiofemoral cartilage loss over 2.6 years	LightGBM	0.845	(0.780,0.910)	0.878	0.714	0.885	0.700	0.881	0.831	**<0.001**
	Logistic regression	0.701	(0.654,0.748)	0.493	0.830	0.899	0.348	0.637	0.576	
Tibial cartilage loss over 10.7 years	LightGBM	0.845	(0.785,0.905)	0.853	0.745	0.884	0.691	0.868	0.820	**0.016**
	Logistic regression	0.753	(0.709,0.797)	0.810	0.599	0.837	0.553	0.823	0.750	
**Onset of knee pain**										
Baseline to 2.6 year	LightGBM	0.813	(0.667,0.960)	0.818	0.851	0.450	0.969	0.581	0.847	0.291
	Logistic regression	0.723	(0.642,0.803)	0.704	0.686	0.345	0.908	0.463	0.689	
Baseline to 5.1 years	LightGBM	0.808	(0.713,0.903)	0.857	0.703	0.486	0.938	0.620	0.741	0.365
	Logistic regression	0.755	(0.691,0.818)	0.827	0.623	0.331	0.941	0.473	0.661	
Baseline to 10.7 years	LightGBM	0.783	(0.639,0.926)	0.727	0.757	0.308	0.949	0.433	0.753	0.258
	Logistic regression	0.689	(0.615,0.763)	0.589	0.748	0.367	0.880	0.452	0.716	
**Progression of knee pain**										
Baseline to 2.6 year	LightGBM	0.775	(0.654,0.897)	0.850	0.582	0.378	0.929	0.523	0.644	0.213
	Logistic regression	0.687	(0.619,0.754)	0.699	0.645	0.398	0.864	0.507	0.659	
Baseline to 5.1 years	LightGBM	0.912	(0.844,0.979)	0.812	0.901	0.650	0.955	0.722	0.885	**<0.001**
	Logistic regression	0.737	(0.662,0.811)	0.525	0.870	0.508	0.877	0.516	0.799	
Baseline to 10.7 years	LightGBM	0.839	(0.739,0.938)	0.750	0.806	0.480	0.931	0.585	0.795	0.121
	Logistic regression	0.739	(0.664,0.815)	0.759	0.690	0.396	0.914	0.520	0.704	
**Onset of functional disability**										
Baseline to 2.6 year	LightGBM	0.812	(0.706,0.918)	0.842	0.643	0.444	0.923	0.581	0.693	**0.018**
	Logistic regression	0.644	(0.554,0.733)	0.540	0.705	0.314	0.860	0.397	0.672	
Baseline to 5.1 years	LightGBM	0.808	(0.650,0.966)	0.889	0.667	0.267	0.978	0.411	0.693	0.267
	Logistic regression	0.706	(0.620,0.792)	0.480	0.855	0.453	0.868	0.466	0.780	
Baseline to 10.7 years	LightGBM	0.786	(0.675,0.897)	0.875	0.610	0.378	0.947	0.528	0.667	0.123
	Logistic regression	0.680	(0.605,0.755)	0.925	0.423	0.304	0.953	0.458	0.530	
**Progression of functional disability**										
Baseline to 2.6 year	LightGBM	0.731	(0.611,0.852)	0.600	0.831	0.480	0.889	0.533	0.784	0.152
	Logistic regression	0.627	(0.552,0.702)	0.803	0.401	0.256	0.888	0.388	0.483	
Baseline to 5.1 years	LightGBM	0.758	(0.646,0.870)	0.750	0.778	0.400	0.940	0.522	0.773	0.543
	Logistic regression	0.717	(0.647,0.786)	0.552	0.796	0.416	0.871	0.474	0.745	
Baseline to 10.7 years	LightGBM	0.836	(0.748,0.924)	0.773	0.775	0.515	0.917	0.618	0.774	**0.007**
	Logistic regression	0.680	(0.611,0.749)	0.646	0.652	0.402	0.836	0.496	0.650	

AUC, area under the curve; CI, confidence interval; NPV, negative predictive value; PPV, positive predictive value; TKR, total knee replacement.

a Due to imbalance of outcomes, using the area under the precision-recall curve as results.

The top ten features of the LightGBM model for the onset and progression of knee symptoms are shown in Supplementary Figs. 3–4. Overall, WOMAC pain and function scores, physical activity, height, weight, tibial cartilage volume, and medial subchondral BMD in the region of interest-1 (ROI-1) at baseline played a significant role in predicting the onset and progression of knee symptoms. The SHAP explanations for model predictions are presented in Supplementary Figs. 5–6.

### 3.3 Predictive validity for tibial cartilage loss

**Fig. 4:**
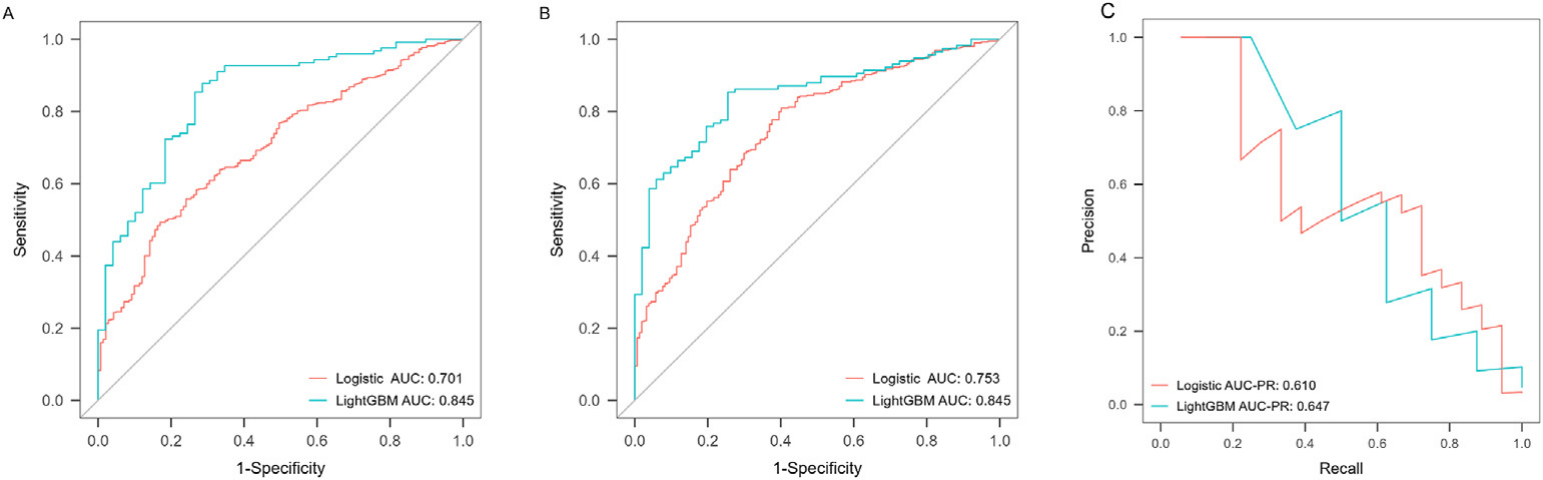
Predictive validity of LightGBM and logistic regression models for the incidence of ≥1 ​%/year loss in tibial cartilage volume over 2.6 (A) and 10.7 (B) years and the risk of TKR (C) over 13.6 years.

The LightGBM model performed significantly better than logistic regression models for tibial cartilage loss over 2.6 (AUC 0.845 vs 0.701, p ​< ​0.001) and 10.7 years (AUC 0.845 vs 0.753, p ​= ​0.016) (Table 2, Fig. 4 (A-B)). Moreover, tibial cartilage volume, weight, lateral and medial subchondral BMD in ROI-2, and medial subchondral BMD in ROI-1 at baseline were the top five important features for predicting tibial cartilage loss over 2.6 years, and tibial cartilage volume, age, weight, BMLs, and physical activity for predicting tibial cartilage loss over 10.7 years (Supplementary Fig. 7). Supplementary Fig. 8 presents the SHAP-based explanations for the model predictions.

### 3.4 Predictive validity for TKR

TKR showed significant imbalance since only 3 ​% cases were identified, for which AUC-PR of both LightGBM and logistic regression model showed relatively low predictive validity (0.647 vs 0.610), Table 2 and Fig. 4 (C)).

In the LightGBM model, JSN was the mainstay for predicting the risk of TKR, followed by BMLs and cartilage defects at baseline (Supplementary Fig. 7). WOMAC pain and function scores and osteophytes did not show great importance in the LightGBM model. The model predictions are explained using SHAP values, as illustrated in Supplementary Fig. 8.

## 4 Discussion

Using data from a community-dwelling older adults cohort, we compared the predictive validity of LightGBM algorithm with logistic regression models that combined the Delphi definition of MRI-OA, ROA, and common covariates for the onset and progression of knee pain and function, tibial cartilage loss, and the risk of TKR. Overall, LightGBM showed a clinically acceptable predictive performance for most outcomes (AUC >0.7). However, the predictive validity of both LightGBM and logistic regression models for the risk of TKR was poor based on AUC-PR, suggesting a limited effectiveness of LightGBM for imbalanced outcomes. Compared to traditional logistic regression models that combined MRI-OA, ROA, and common covariates, LightGBM performed better in predicting not only the onset and progression of knee pain and function over 2.6, 5.1, and 10.7 years but also a clinically important reduction in tibial cartilage volume (i.e. ≥1 ​%/year) over 2.6 and 10.7 years, although not all metrics of LightGBM models performed well (e.g. PPV, F1 and AUC-PR were relatively low). Overall, these findings suggest that an ML-based data-driven approach can offers valuable insights for early risk identification and targeted prevention strategies.

The prediction of knee OA progression poses challenges attributed to the heterogeneous and multi-factorial character of the disease. A recent systematic review has indicated the importance of combining structural and clinical factors for the prediction of radiographic progression of knee OA [27]. Our previous study also illustrated a significantly greater strength of combining MRI-OA with ROA for predicting structural and symptomatic progression of knee OA [5]. However, factors used to build predictive models are commonly derived from epidemiological evidence for their potential role in the progression of OA, whereas the effect of other covariables may be unclear and not be included in predictive models. In contrast, ML models include as many factors that are related or unrelated to the outcome of interest based on existing knowledge. While logistic regression models may be more interpretable and provide insights into well-established risk factors, ML models have the advantage of potentially uncovering novel and less explored predictors. Overall, our findings suggest that the LightGBM model holds great potential for predicting the progression of KOA and can be well-suited for early screening in clinical practice.

In this study, we found that the LightGBM were superior to traditional logistic regression models for predicting the onset and progression of knee pain and functional disability over 2.6–10.7 years. In a recent study, a traditional OA risk assessment model combined with a deep learning approach, a subset of ML, showed good predictive validity for the progression of knee pain over 4 years (AUC: 0.81) [28]. Recently, Lin et al. [29] introduced a dynamic nomogram based on MRI-derived radiomics for predicting knee pain improvement over a two-year period in individuals with KOA. Loos et al. [30] conducted ML methods to forecast post-surgery function and pain outcomes for thumb carpometacarpal OA. These studies suggest that, for knee symptoms, ML models have great potential in screening high-risk individuals and may be used in clinical practice. Under the situation of the suboptimal implementation of guidelines for patients with knee OA [31], unfavorable results from prediction models can serve as a valuable incentive for both patients and clinicians to proactively implement the recommended therapies such as exercise and weight control [32,33].

For tibial cartilage volume loss (≥ or ​< ​1 ​%/year) over 2.6 and 10.7 years, LightGBM models showed similar predictive validity for both time intervals (AUC: 0.845 and 0.845) and were significantly better than logistic regression models (AUC: 0.701 and 0.753). In the LightGBM, we found that tibial cartilage volume at baseline was, as expected, the key predictor of cartilage loss over 2.6 and 10.7 years, followed by weight for both time intervals, suggesting a critical impact of mechanical loading on the structural progression of knee OA. Interestingly, subchondral BMD and BMLs predicted tibial cartilage loss over 2.6 and 10.7 years, respectively, providing further support that subchondral bone abnormalities play an important role in the progression of knee OA over both short- and long-term. Age and physical activity (i.e. >1000 steps/day) predicted tibial cartilage loss over 10.7 years. We previously showed that tibial cartilage loss over 10.7 years was greater with increasing age [34]. However, research investigating the influence of physical activity on cartilage loss has produced conflicting results [[35], [36], [37]]. In this study, although the LightGBM models showed that physical activity as an important predictor of tibial cartilage loss over 10.7 years, we cannot be sure whether it accelerated or slowed the process.

LightGBM performed worse than logistic regression models for the risk of TKR, which may be due to the low positive rate of the outcome. The high predictive validity of the logistic regression models may be due to that both knee symptoms and ROA features were included in the model, and they have been shown to be the main driver for TKR [38]. Consistently, the predictive validity and top predictors in our study are comparable to other ML-based predictive models for the risk of TKR over 2–5 years [8,39,40].

In this study, LightGBM had moderate-to-high sensitivity and specificity while low PPV and F1 for most outcomes of interest. In the lack of an established definition for early-stage KOA [41], the predictive model still has some value, especially for screening purposes. The high sensitivity enables the identification of high-risk individuals at the early stages, even before significant symptoms develop, allowing timely lifestyle modifications, therapeutic interventions, and delayed progression to severe outcomes like TKR. However, its low PPV can result in over-diagnosis, unnecessary treatments, and increased healthcare costs. Therefore, future predictive models are warranted to improve PPV by reducing false positives or incorporating additional clinical factors would enhance its clinical utility and reduce the risk of over-treatment.

One of the key strengths of our study lies in its population-based approach and its extensive long-term follow-up period. Moreover, we compared the predictive validity of LightGBM with logistic regression models that combined MRI-OA, ROA, and common covariates, which have been previously demonstrated to have strong predictive validity for the progression of knee OA [5]. Our study has several limitations. First, as discussed above, any difference in terms of predictive validity may be due to the differences in covariables included. However, rather than to identify key predictors, the main aim of this study was to determine which models, based on their standard practice, would better predict the progression of knee OA. Second, the sample size of this study was modest and the lack of external testing in independent cohorts may restrict the generalizability of our finding. Differences in MRI feature measurements across public cohorts (e.g., MOST and OAI) hindered the inclusion of external data. Nonetheless, we employed rigorous internal validation through cross-validation to mitigate the risk of overfitting and provide reliable performance estimates. These methods are widely recognized in predictive modeling literature as robust indicators of model performance, especially when external datasets with comparable characteristics and follow-up are unavailable. Future external validation will be needed to further enhance the generalizability of our findings. Third, we only evaluated one ML method (LightGBM) and compared its predictive validity with traditional logistic regression models. However, a previous study has shown that the ML methods had similar performance and the LightGBM had the highest accuracy in predicting the risk of TKR [8].

In conclusion, compared to logistic regression models that combined the Delphi definition of MRI-OA, ROA, and common covariates, LightGBM model incorporating individual features could offer valuable insights that can inform early risk identification and targeted prevention strategies.

## Contributors

The guarantor (GC) had full access to all of the data in the study and took responsibility for the integrity of the data and the accuracy of the data analysis. GC conceived, initiated, and supervised the project. XX and GC cleaned and analyzed the data. All authors contributed to the interpretation of the results and writing and revision of the manuscript. All authors gave final approval of the version submitted. The corresponding author attests that all listed authors meet authorship criteria and that no others meeting the criteria have been omitted.

## Ethics approval

Southern Tasmanian Health and Medical Human Research Ethics Committee.

## Data availability statement

The data that support the findings of this study are available from the corresponding author upon reasonable request. All of the code in this study was made available on the Open Science Framework (https://osf.io/qwtue/).

## Funding sources

This work is supported by the 10.13039/501100001809National Natural Science Foundation of China (82473719, 82103933), Research Innovation Team Project of School of Public Health, 10.13039/501100002947Anhui Medical University (kctd200403), and the Scientific Research Level Upgrading Project of 10.13039/501100002947Anhui Medical University (2021xkjT006).

## Declaration of competing interest

None.
