# Supplementary material for: Predictive validity of consensus-based MRI definition of osteoarthritis plus radiographic osteoarthritis for the progression of knee osteoarthritis: A longitudinal cohort study
Source: Osteoarthr Cartil Open. 2025 Feb 15;7(2):100582. doi: 10.1016/j.ocarto.2025.100582 (PMC11889593; doi:10.1016/j.ocarto.2025.100582)
Supplement: Multimedia component 1 [file mmc1.docx]

**Supplementary File**

Supplementary Table 1. Predictors in ML models

Supplementary Table 2. Summary information of missing data in predictors

Supplementary Table 3. The results of the cross-validation loop

Supplementary Table 4. The adjusted hyperparameters and parameters in LightGBM

Supplementary Table 5. The proportion of positive results of each outcome

Supplementary Figure 1. Study flowchart

Supplementary Figure 2. Directed acyclic graph (DAG) of covariates

Supplementary Figure 3. Feature importance for LightGBM models for prediction of progression of symptoms in pain at 2.6 (A), 5.1 (B) and 10.7 (C) years and onset of symptoms in pain at 2.6 (D), 5.1 (E) and 10.7 (F) years

Supplementary Figure 4. Feature importance for LightGBM models for prediction of progression of functional disability at 2.6 (A), 5.1 (B) and 10.7 (C) years and onset of functional disability at 2.6 (D), 5.1 (E) and 10.7 (F) years

Supplementary Figure 5. SHAP for LightGBM models for prediction of progression of symptoms in pain at 2.6 (A), 5.1 (B) and 10.7 (C) years and onset of symptoms in pain at 2.6 (D), 5.1 (E) and 10.7 (F) years

Supplementary Figure 6. SHAP for LightGBM models for prediction of progression of functional disability at 2.6 (A), 5.1 (B) and 10.7 (C) years and onset of functional disability at 2.6 (D), 5.1 (E) and 10.7 (F) years

Supplementary Figure 7. Feature importance of LightGBM models for prediction of incidence of tibial cartilage volume loss at 2.6 (A), 10.7 (B) years and TKR over 13.5 years(C)

Supplementary Figure 8. SHAP of LightGBM models for prediction of incidence of tibial cartilage volume loss at 2.6 (A) and 10.7 (B) years and TKR over 13.5 years(C)

Supplementary Table 1. Predictors in ML models

| Models | | Model 1: risk of total knee replacement over 13.5 year | | Model 2: Risk of 1%/year loss in tibial cartilage volume over 2.6 year | | Model 3: Risk of 1%/year loss in tibial cartilage volume over 10.7 year | | Model 4-15: Predicting the onset and progression of knee pain and Function disability over 2.6, 5.1, and 10.7 years |  |
| --- | --- | --- | --- | --- | --- | --- | --- | --- | --- |
| Demographic | Demographic | Gender | | Gender | | Gender | | Gender |  |
|  |  | Age | | Age | | Age | | Age |  |
|  |  | Height | | Height | | Height | | Height |  |
|  |  | BMI^[[1]](#endnote-1)^ | | BMI | | BMI | | BMI |  |
|  |  | Weight | | Weight | | Weight | | Weight |  |
|  | Socioeconomic | Socioeconomic status (IRSD) | | Socioeconomic status (IRSD) | | Socioeconomic status (IRSD) | | Socioeconomic status (IRSD) |  |
|  |  | Socioeconomic status (IRSAD) | | Socioeconomic status (IRSAD) | | Socioeconomic status (IRSAD) | | Socioeconomic status (IRSAD) |  |
|  |  | Socioeconomic status (IER) | | Socioeconomic status (IER) | | Socioeconomic status (IER) | | Socioeconomic status (IER) |  |
|  |  | Socioeconomic status (IEO) | | Socioeconomic status (IEO) | | Socioeconomic status (IEO) | | Socioeconomic status (IEO) |  |
|  |  | Smoke | | Smoke | | Smoke | | Smoke |  |
|  |  | Current Employment | | Current Employment | | Current Employment | | Current Employment |  |
|  |  | Quality of life | | Quality of life | | Quality of life | | Quality of life |  |
|  |  | physical activity | | physical activity | | physical activity | | physical activity |  |
| Medical History | OA-related | History of knee injury | | History of knee injury | | History of knee injury | | History of knee injury |  |
|  |  | History of hip pain | | History of hip pain | | History of hip pain | | History of hip pain |  |
|  |  | History of knee surgery | | History of knee surgery | | History of knee surgery | | History of knee surgery |  |
|  | Comorbidities | Any comorbidity | | Any comorbidity | | Any comorbidity | | Any comorbidity |  |
|  |  | Consumed any pain-relief medication | | Consumed any pain-relief medication | | Consumed any pain-relief medication | | Consumed any pain-relief medication |  |
|  | Clinical  examination | WOMAC^[[2]](#endnote-2)^ pain score | | WOMAC pain score | | WOMAC pain score | | WOMAC pain score |  |
|  |  | WOMAC stiffness score | | WOMAC stiffness score | | WOMAC stiffness score | | WOMAC stiffness score |  |
|  |  | WOMAC Function score | | WOMAC Function score | | WOMAC Function score | | WOMAC Function score |  |
| Radiograph | | JSN^[[3]](#endnote-3)^ score | JSN score | | JSN score | | JSN score | |  |
|  |  | Osteophytes of the medial and lateral | | Osteophytes of the medial and lateral | | Osteophytes of the medial and lateral | | Osteophytes of the medial and lateral | |
| MRI features | Cartilage | Tibial cartilage volume in Wave1 (baseline), mm3, Wave1&2 unpaired measurements | | Tibial cartilage volume in Wave1 (baseline), mm3, Wave1&2 unpaired measurements | | Tibial cartilage volume in Wave1 (baseline), mm3, Wave1&4 paired measurements | | Tibial cartilage volume in Wave1 (baseline), mm3, Wave1&2 unpaired measurements |  |
|  |  | Femoral cartilage volume in Wave1 (baseline), mm3, Wave1&2 unpaired measurements | | Femoral cartilage volume in Wave1 (baseline), mm3, Wave1&2 unpaired measurements | |  |  | Femoral cartilage volume in Wave1 (baseline), mm3, Wave1&2 unpaired measurements |  |
|  |  | Total bone area | | Total bone area | | Total bone area | | Total bone area |  |
|  |  | Medial tibial cartilage defects | | Medial tibial cartilage defects | | Medial tibial cartilage defects | | Medial tibial cartilage defects |  |
|  |  | Medial femoral cartilage defects | | Medial femoral cartilage defects | | Medial femoral cartilage defects | | Medial femoral cartilage defects |  |
|  |  | Lateral tibial cartilage defects | | Lateral tibial cartilage defects | | Lateral tibial cartilage defects | | Lateral tibial cartilage defects |  |
|  |  | Lateral femoral cartilage defects | | Lateral femoral cartilage defects | | Lateral femoral cartilage defects | | Lateral femoral cartilage defects |  |
|  | Bone mineral density | Standardized medical subchondral bone mineral density in ROI^[[4]](#endnote-4)^ 1 | | Standardized medical subchondral bone mineral density in ROI 1 | | Standardized medical subchondral bone mineral density in ROI 1 | | Standardized medical subchondral bone mineral density in ROI 1 |  |
|  |  | Standardized medical subchondral bone mineral density in ROI 2 | | Standardized medical subchondral bone mineral density in ROI 2 | | Standardized medical subchondral bone mineral density in ROI 2 | | Standardized medical subchondral bone mineral density in ROI 2 |  |
|  |  | Standardized medical subchondral bone mineral density in ROI 3 | | Standardized medical subchondral bone mineral density in ROI 3 | | Standardized medical subchondral bone mineral density in ROI 3 | | Standardized medical subchondral bone mineral density in ROI 3 |  |
|  |  | Standardized lateral subchondral bone mineral density in ROI 1 | | Standardized lateral subchondral bone mineral density in ROI 1 | | Standardized lateral subchondral bone mineral density in ROI 1 | | Standardized lateral subchondral bone mineral density in ROI 1 |  |
|  |  | Standardized lateral subchondral bone mineral density in ROI 2 | | Standardized lateral subchondral bone mineral density in ROI 2 | | Standardized lateral subchondral bone mineral density in ROI 2 | | Standardized lateral subchondral bone mineral density in ROI 2 |  |
|  |  | Standardized lateral subchondral bone mineral density in ROI 3 | | Standardized lateral subchondral bone mineral density in ROI 3 | | Standardized lateral subchondral bone mineral density in ROI 3 | | Standardized lateral subchondral bone mineral density in ROI 3 |  |
|  | Other | Total cartilage defects | | Total cartilage defects | | Total cartilage defects | | Total cartilage defects |  |
|  |  | Total meniscus lesion score | | Total meniscus lesion score | | Total meniscus lesion score | | Total meniscus lesion score |  |
|  |  | Total bone marrow lesion size | | Total bone marrow lesion size | | Total bone marrow lesion size | | Total bone marrow lesion size |  |
|  |  | Total effusion synovitis | | Total effusion synovitis | | Total effusion synovitis | | Total effusion synovitis |  |
|  |  | MRI^[[5]](#endnote-5)^-defined osteophytes | | MRI-defined osteophytes | | MRI-defined osteophytes | | MRI-defined osteophytes |  |

| Variable | Proportion of missing | Variable | Proportion of missing |
| --- | --- | --- | --- |
| WOMAC^[[6]](#endnote-6)^ pain score in Wave 1 | 0.00% | Total meniscus lesion score | 4.53% |
| WOMAC stiffness score in Wave 1 | 0.17% | Total bone marrow lesion size | 6.45% |
| WOMAC function score in Wave 1 | 0.00% | Total effusion synovitis | 0.17% |
| WOMAC pain score in Wave 2 | 2.96% | Physical activity | 2.09% |
| WOMAC stiffness score in Wave 2 | 2.96% | MRI^[[7]](#endnote-7)^-defined osteophytes | 0.35% |
| WOMAC function score in Wave 2 | 2.96% | Consumed any pain-relief medication | 0.00% |
| WOMAC pain score in Wave 3 | 11.67% | Hip pain History | 1.22% |
| WOMAC stiffness score in Wave 3 | 11.67% | Any comorbidity | 0.00% |
| WOMAC function score in Wave 3 | 11.67% | Quality of life in Baseline | 0.00% |
| WOMAC pain score in Wave 4 | 26.66% | Knee ROA^[[8]](#endnote-8)^ | 0.00% |
| WOMAC stiffness score in Wave 4 | 26.83% | MRI OA^[[9]](#endnote-9)^ | 0.00% |
| WOMAC function score in Wave 4 | 26.66% | MRI_ROA | 0.00% |
| BMI^[[10]](#endnote-10)^ in Baseline | 0.00% | No TKR in Wave 2 | 0.00% |
| TKR^[[11]](#endnote-11)^ | 0.00% | No TKR in Wave3 | 0.00% |
| Socioeconomic status by IRSAD | 2.26% | Medial tibial cartilage defects | 0.00% |
| Socioeconomic status by IER | 2.26% | Medial femoral cartilage defects | 0.00% |
| Total bone area | 8.01% | Lateral tibial cartilage defects | 0.00% |
| Socioeconomic status by IEO | 2.26% | No TKR in Wave4 | 0.00% |
| Socioeconomic status by IRSD | 2.26% | OA | 0.00% |
| Knee Surgery History | 0.52% | Current Employment | 0.00% |
| Gender | 0.00% | Smoke | 0.17% |
| Standardized medical subchondral bone mineral density in ROI^[[12]](#endnote-12)^ 1 | 37.80% | Annual percentage change in tibiofemoral cartilage volume | 50.87% |
| Standardized medical subchondral bone mineral density in ROI 2 | 37.80% | Tibial cartilage volume in Wave1 (baseline), mm3, Wave1&4 paired measurements | 34.32% |
| Standardized medical subchondral bone mineral density in ROI 3 | 33.28% | Tibial cartilage volume in Wave1 (baseline), mm3, Wave1&2 unpaired measurements | 0.52% |
| Standardized lateral subchondral bone mineral density in ROI 1 | 37.80% | Tibial cartilage volume in Wave1 (baseline), mm3, Wave1&4 paired measurements | 38.85% |
| Standardized lateral subchondral bone mineral density in ROI 3 | 33.28% | Femoral cartilage volume in Wave1 (baseline), mm3, Wave1&2 unpaired measurements | 5.57% |
| Annual percentage change in tibial cartilage volume^[[13]](#endnote-13)^ | 34.32% | Standardized lateral subchondral bone mineral density in ROI 2 | 37.80% |
| Height | 0.00% | Knee Injury History | 3.14% |
| Weight | 0.00% | osteophytes of the medial and lateral | 0.00% |
| Age | 0.00% | Total cartilage defects | 0.00% |
| JSN^[[14]](#endnote-14)^ score | 38.80% | Current Employment | 0.00% |
| Lateral femoral cartilage defects | 0.17% |  |  |

Supplementary Table 2. Summary information of missing data in predictor

Supplementary Table 3. The results of the cross-validation loop

| **Outcomes** | | **fold1** | **fold2** | **fold3** | **fold4** | **fold5** | **Mean** |
| --- | --- | --- | --- | --- | --- | --- | --- |
| **Incidence of TKR over 13.5 years** | | 0.632 | 0.619 | 0.611 | 0.649 | 0.638 | 0.63 |
| **Tibiofemoral cartilage loss over 2.6 years** | | 0.836 | 0.856 | 0.851 | 0.848 | 0.842 | 0.847 |
| **Tibial cartilage loss over 10.7 years** | | 0.853 | 0.851 | 0.841 | 0.839 | 0.831 | 0.843 |
| **Onset of knee pain** | Baseline to 2.6 year | 0.799 | 0.81 | 0.801 | 0.829 | 0.819 | 0.812 |
|  | Baseline to 5.1 years | 0.815 | 0.802 | 0.793 | 0.794 | 0.809 | 0.803 |
|  | Baseline to 10.7 years | 0.767 | 0.796 | 0.801 | 0.794 | 0.799 | 0.791 |
| **Progression of knee pain** | Baseline to 2.6 year | 0.789 | 0.781 | 0.792 | 0.762 | 0.774 | 0.78 |
|  | Baseline to 5.1 years | 0.901 | 0.912 | 0.916 | 0.923 | 0.911 | 0.913 |
|  | Baseline to 10.7 years | 0.823 | 0.817 | 0.827 | 0.829 | 0.852 | 0.83 |
| **Onset of functional disability** | Baseline to 2.6 year | 0.801 | 0.828 | 0.834 | 0.818 | 0.803 | 0.817 |
|  | Baseline to 5.1 years | 0.8 | 0.803 | 0.806 | 0.817 | 0.812 | 0.808 |
|  | Baseline to 10.7 years | 0.763 | 0.777 | 0.782 | 0.789 | 0.794 | 0.781 |
| **Progression of functional disability** | Baseline to 2.6 year | 0.755 | 0.746 | 0.724 | 0.731 | 0.727 | 0.737 |
|  | Baseline to 5.1 years | 0.759 | 0.768 | 0.747 | 0.749 | 0.75 | 0.755 |
|  | Baseline to 10.7 years | 0.838 | 0.842 | 0.832 | 0.831 | 0.822 | 0.833 |
|  |  |  |  |  |  |  |  |

Supplementary Table 4. The adjusted hyperparameters and parameters in LightGBM

| **Outcomes** | | **num_boost_round** **(1 to 150)** | **learning_rate**  **(1e-3 to 0.6)** | **max_depth**  **(1 to 50)** | **Subsample**  **(0.5 to 1)** | **colsample_bytree**  **(0.5 to 1)** | **reg_alpha**  **(0 to 1000)** | **reg_lambda**  **(0 to 1000)** | **scale_pos_weight**  **(0.1 to 30)** |
| --- | --- | --- | --- | --- | --- | --- | --- | --- | --- |
| **Incidence of TKR over 13.5 years** | | 9 | 0.01 | 8 | 1 | 0.8 | 1 | 0.01 | 4 |
| **Tibiofemoral cartilage loss over 2.6 years** | | 96 | 0.3 | 13 | 0.6 | 0.6 | 5 | 0.5 | 0.3 |
| **Tibial cartilage loss over 10.7 years** | | 27 | 0.2 | 28 | 0.8 | 0.9 | 0.5 | 1 | 0.4 |
| **Onset of knee pain** | Baseline to 2.6 year | 96 | 0.5 | 11 | 0.5 | 0.8 | 0.01 | 0 | 4 |
|  | Baseline to 5.1 years | 62 | 0.6 | 3 | 1 | 0.5 | 0.001 | 0.05 | 5 |
|  | Baseline to 10.7 years | 5 | 0.4 | 25 | 0.7 | 0.5 | 10 | 5 | 3 |
| **Progression of knee pain** | Baseline to 2.6 year | 13 | 0.5 | 2 | 0.5 | 0.7 | 0.01 | 0.05 | 3 |
|  | Baseline to 5.1 years | 100 | 0.5 | 46 | 0.9 | 0.5 | 5 | 50 | 4 |
|  | Baseline to 10.7 years | 47 | 0.5 | 29 | 1 | 0.5 | 1 | 0.01 | 4 |
| **Onset of functional disability** | Baseline to 2.6 year | 8 | 0.6 | 28 | 0.7 | 0.5 | 1 | 0.5 | 4 |
|  | Baseline to 5.1 years | 26 | 0.001 | 29 | 0.9 | 0.5 | 0 | 1 | 3 |
|  | Baseline to 10.7 years | 43 | 0.4 | 32 | 0.7 | 0.9 | 0.001 | 0.1 | 4 |
| **Progression of functional disability** | Baseline to 2.6 year | 8 | 0.4 | 40 | 1 | 0.6 | 0.01 | 0.1 | 4 |
|  | Baseline to 5.1 years | 14 | 0.2 | 27 | 1 | 0.7 | 0.5 | 0.01 | 3 |
|  | Baseline to 10.7 years | 76 | 0.5 | 11 | 0.7 | 0.6 | 0.05 | 1 | 3 |
|  |  |  |  |  |  |  |  |  |  |

Supplementary Table 5. The proportion of positive results of each outcome

| **Outcomes** | | **Proportion** |
| --- | --- | --- |
| **TKR over 13.5 years** | | 0.031 |
| **Tibiofemoral cartilage loss over 2.6 years** | | 0.304 |
| **Tibial cartilage loss over 10.7 years** | | 0.417 |
| **Onset of knee pain** | Baseline to 2.6 year | 0.191 |
|  | Baseline to 5.1 years | 0.194 |
|  | Baseline to 10.7 years | 0.199 |
| **Progression of knee pain** | Baseline to 2.6 year | 0.252 |
|  | Baseline to 5.1 years | 0.254 |
|  | Baseline to 10.7 years | 0.262 |
| **Onset of functional disability** | Baseline to 2.6 year | 0.200 |
|  | Baseline to 5.1 years | 0.220 |
|  | Baseline to 10.7 years | 0.235 |
| **Progression of functional disability** | Baseline to 2.6 year | 0.204 |
|  | Baseline to 5.1 years | 0.208 |
|  | Baseline to 10.7 years | 0.265 |
|  |  |  |


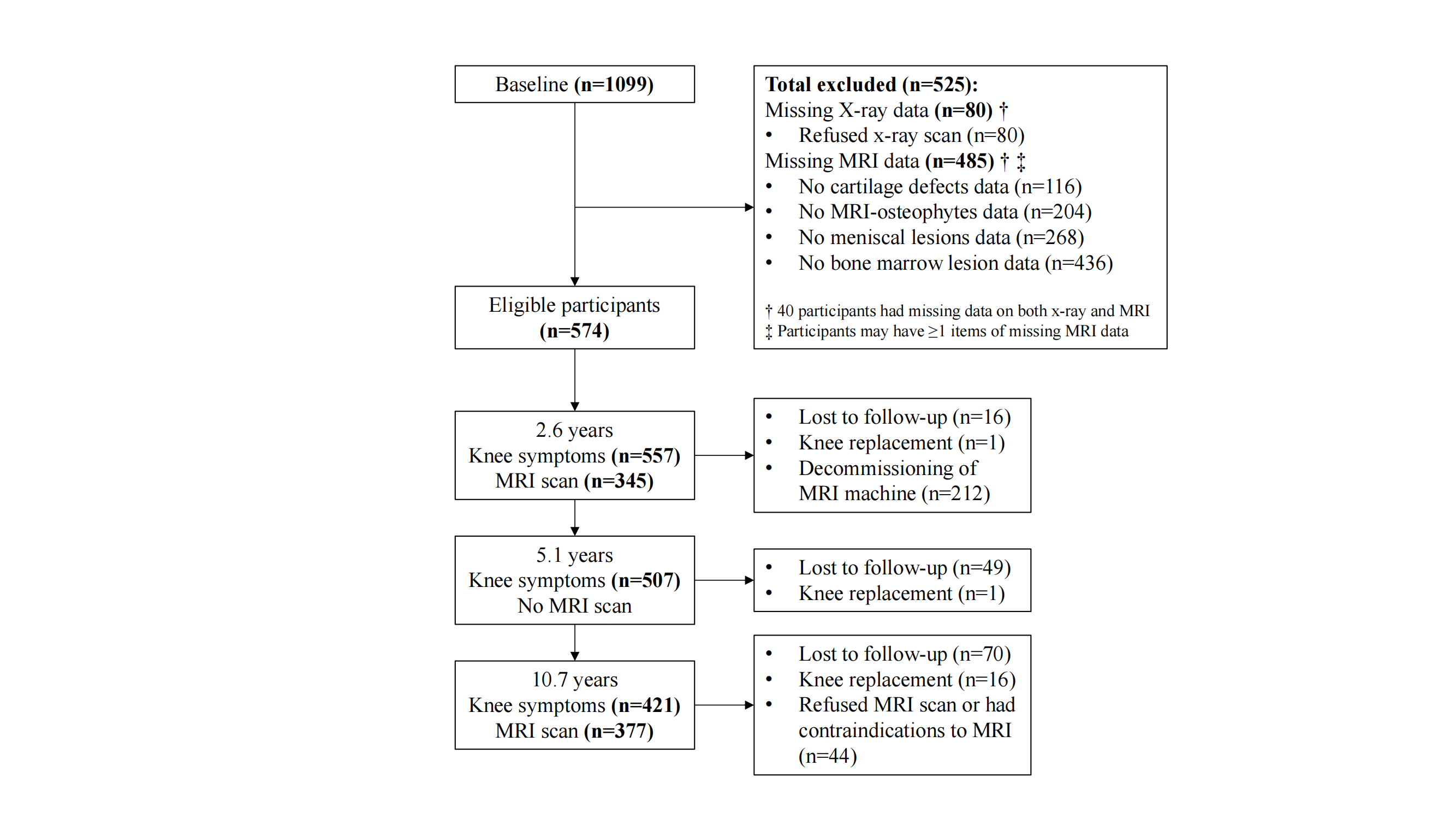
Supplementary Figure 1. Study flowchart

Supplementary Figure 2. Directed acyclic graph (DAG) of covariates


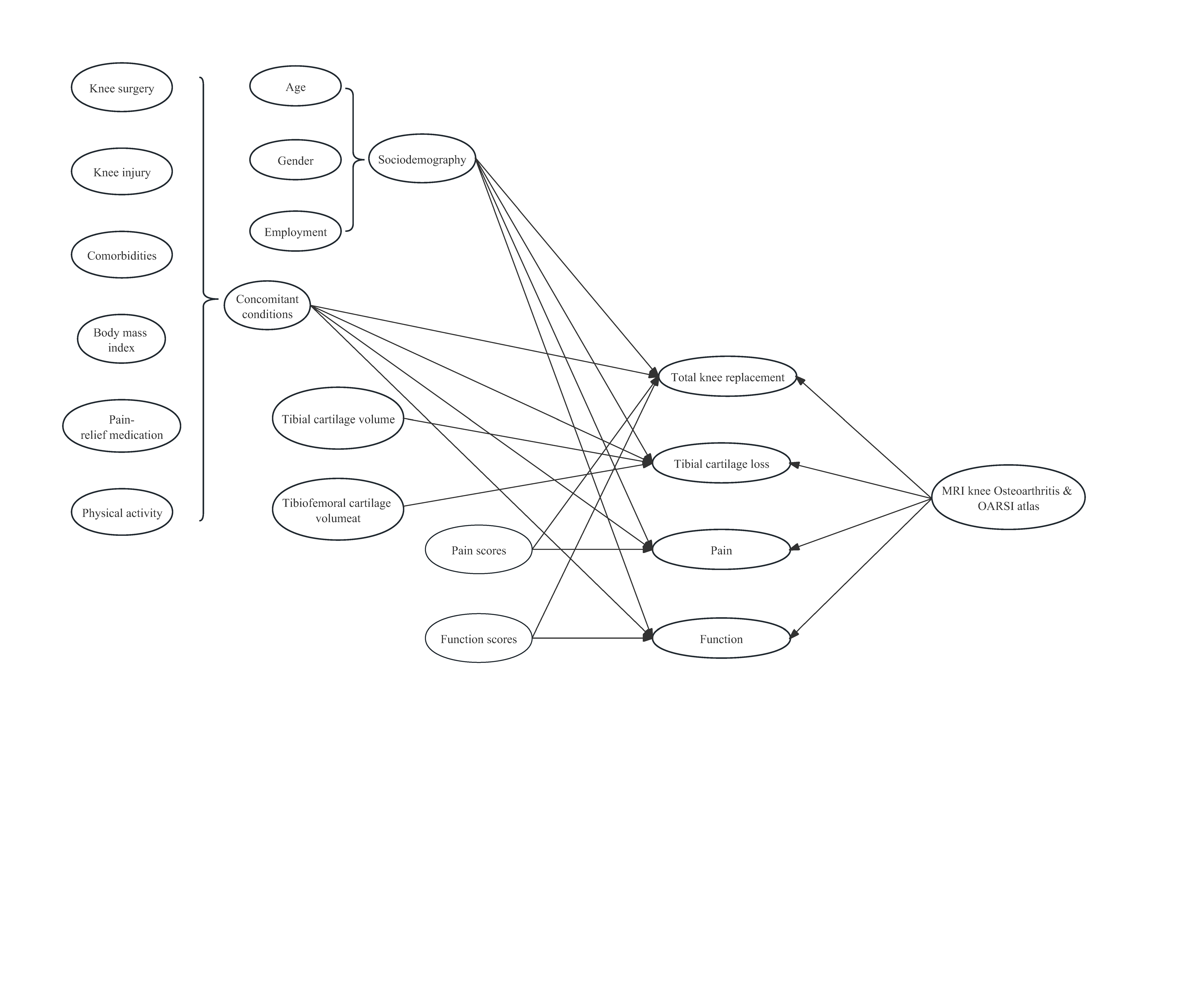


MRI: magnetic resonance imaging; ORASI: Osteoarthritis Research Society International, include joint space narrowing (JSN) grade and osteoporosis (OP) grade.


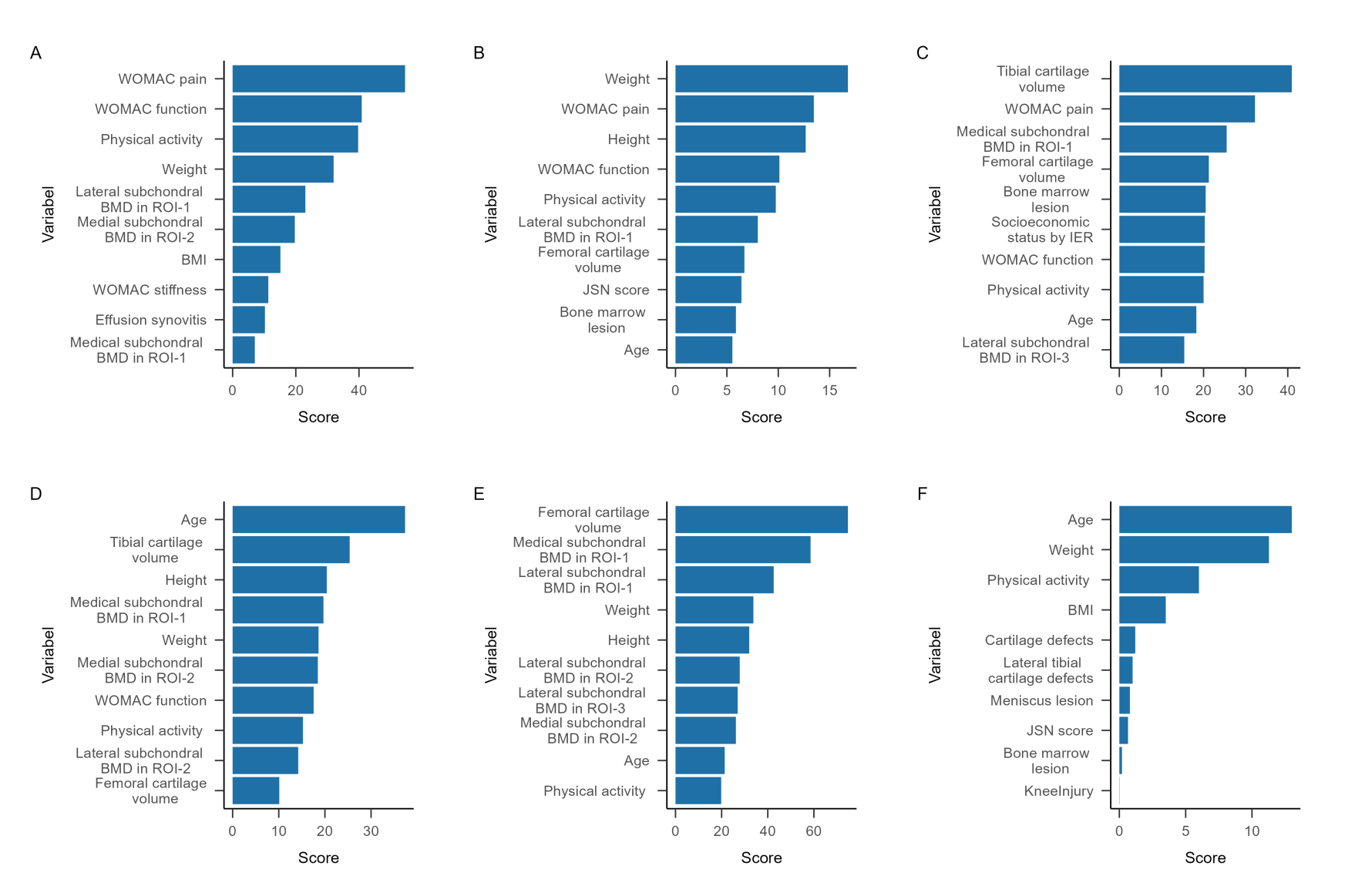
Supplementary Figure 3. Feature importance for lightGBM models for prediction of progression of symptoms in pain at 2.6 (A), 5.1 (B) and 10.7 (C) years and onset of symptoms in pain at 2.6 (D), 5.1 (E) and 10.7 (F) years


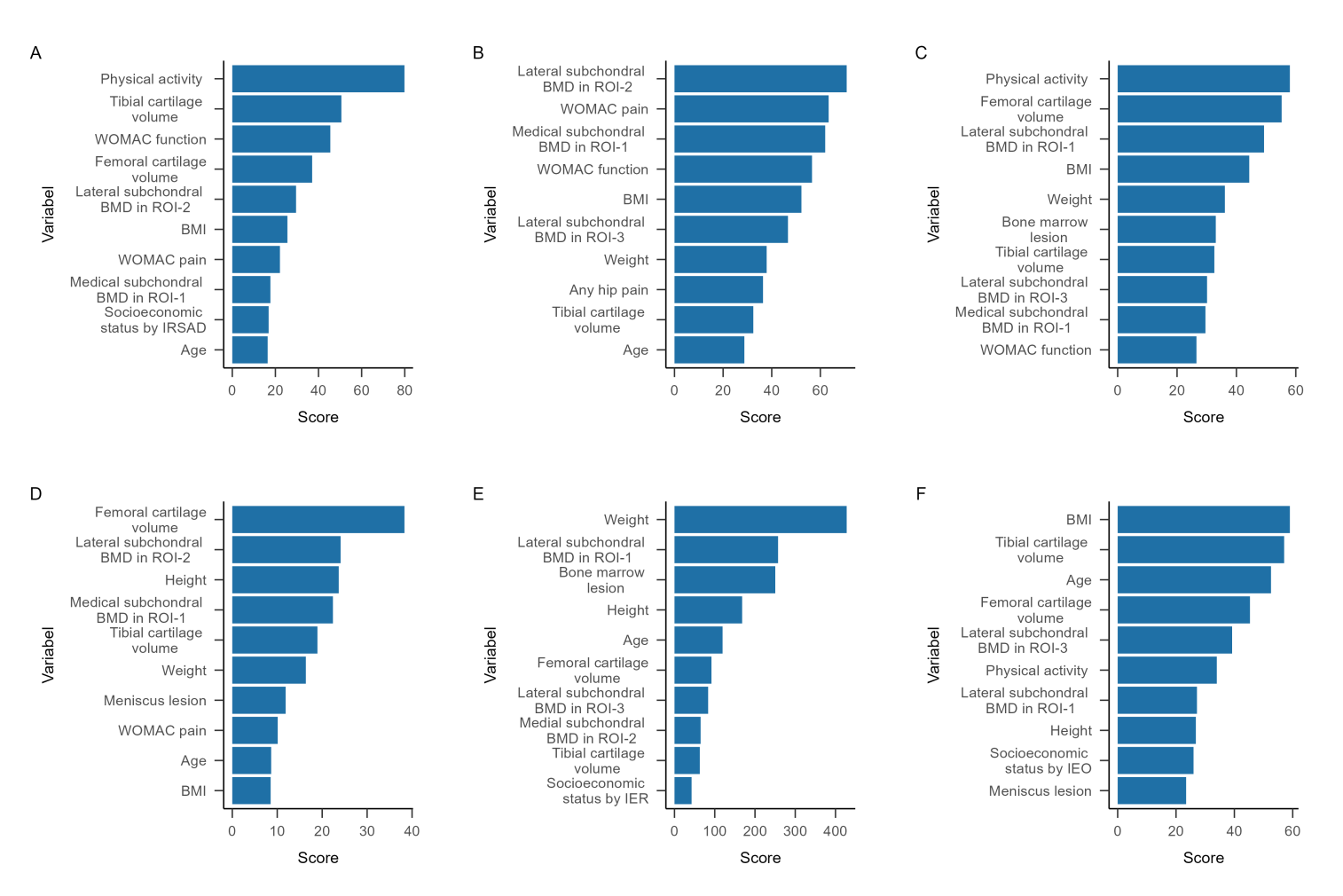
Supplementary Figure 4. Feature importance for lightGBM models for prediction of progression of functional disability at 2.6 (A), 5.1 (B) and 10.7 (C) years and onset of functional disability at 2.6 (D), 5.1 (E) and 10.7 (F) years

Supplementary Figure 5. SHAP for lightGBM models for prediction of progression of symptoms in pain at 2.6 (A), 5.1 (B) and 10.7 (C) years and onset of symptoms in pain at 2.6 (D), 5.1 (E) and 10.7 (F) years


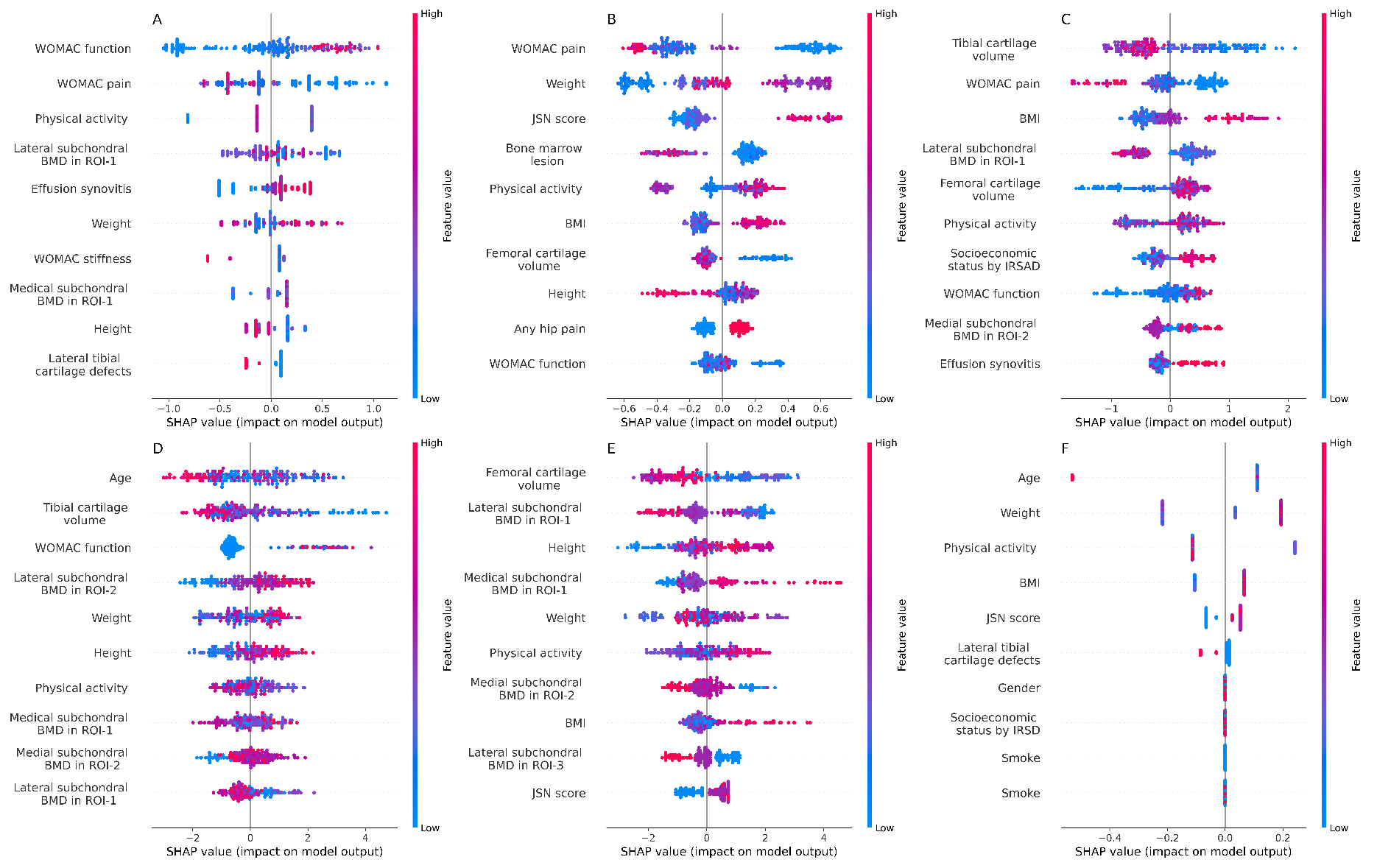


Supplementary Figure 6. SHAP for lightGBM models for prediction of progression of functional disability at 2.6 (A), 5.1 (B) and 10.7 (C) years and onset of functional disability at 2.6 (D), 5.1 (E) and 10.7 (F) years


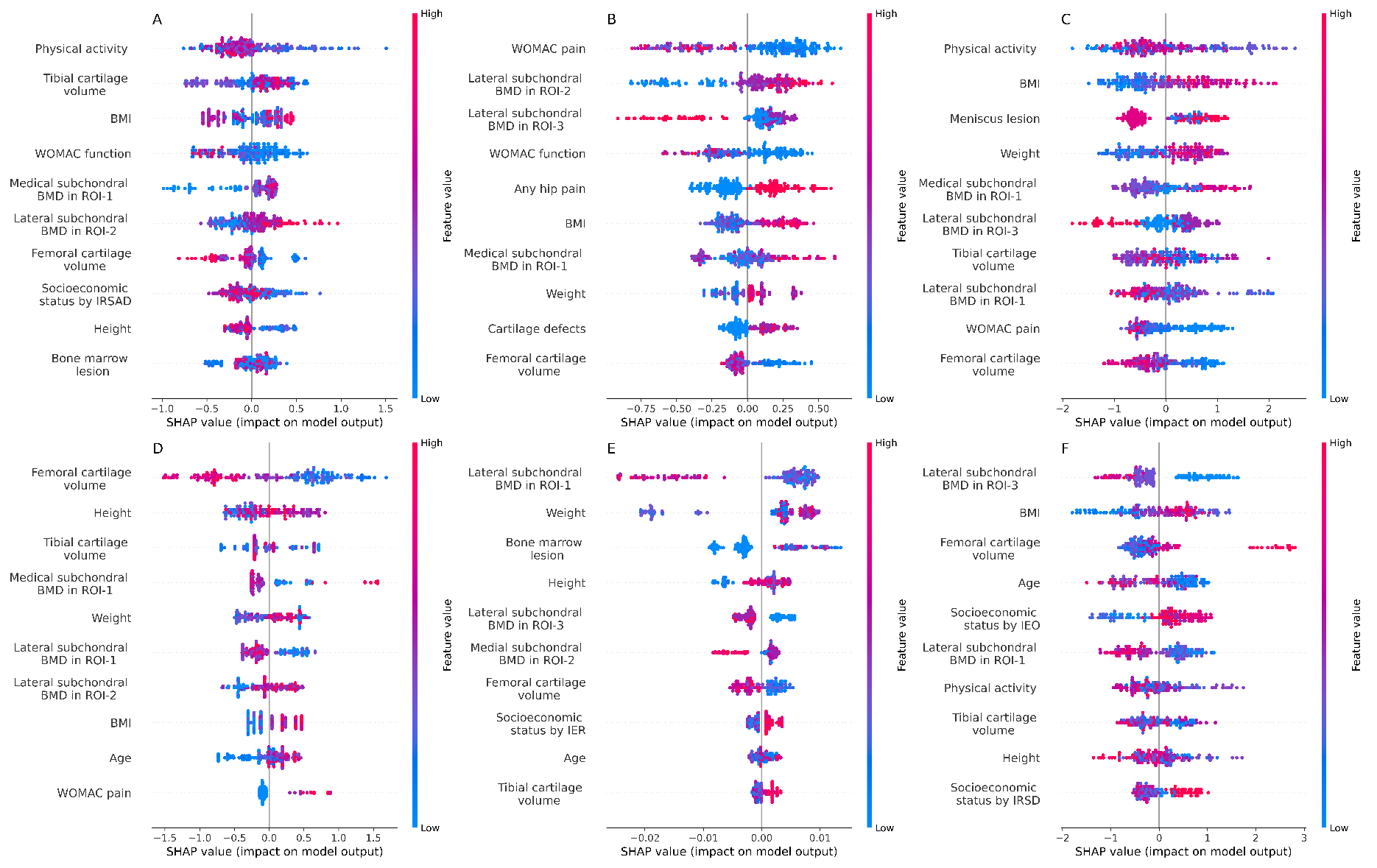


Supplementary Figure 7. Feature importance of lightGBM models for prediction of incidence of tibial cartilage volume loss at 2.6 (A), 10.7 (B) years and TKR over 13.5 years(C)


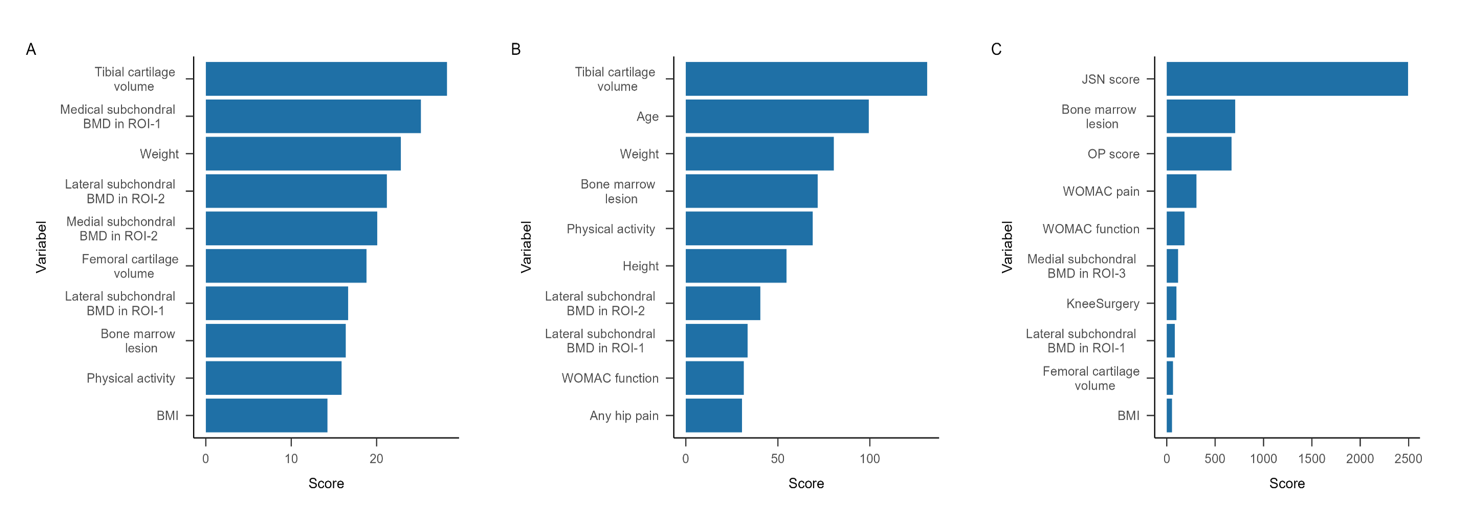


Supplementary Figure 8. SHAP of lightGBM models for prediction of incidence of tibial cartilage volume loss at 2.6 (A) and 10.7 (B) years and TKR over 13.5 years(C)


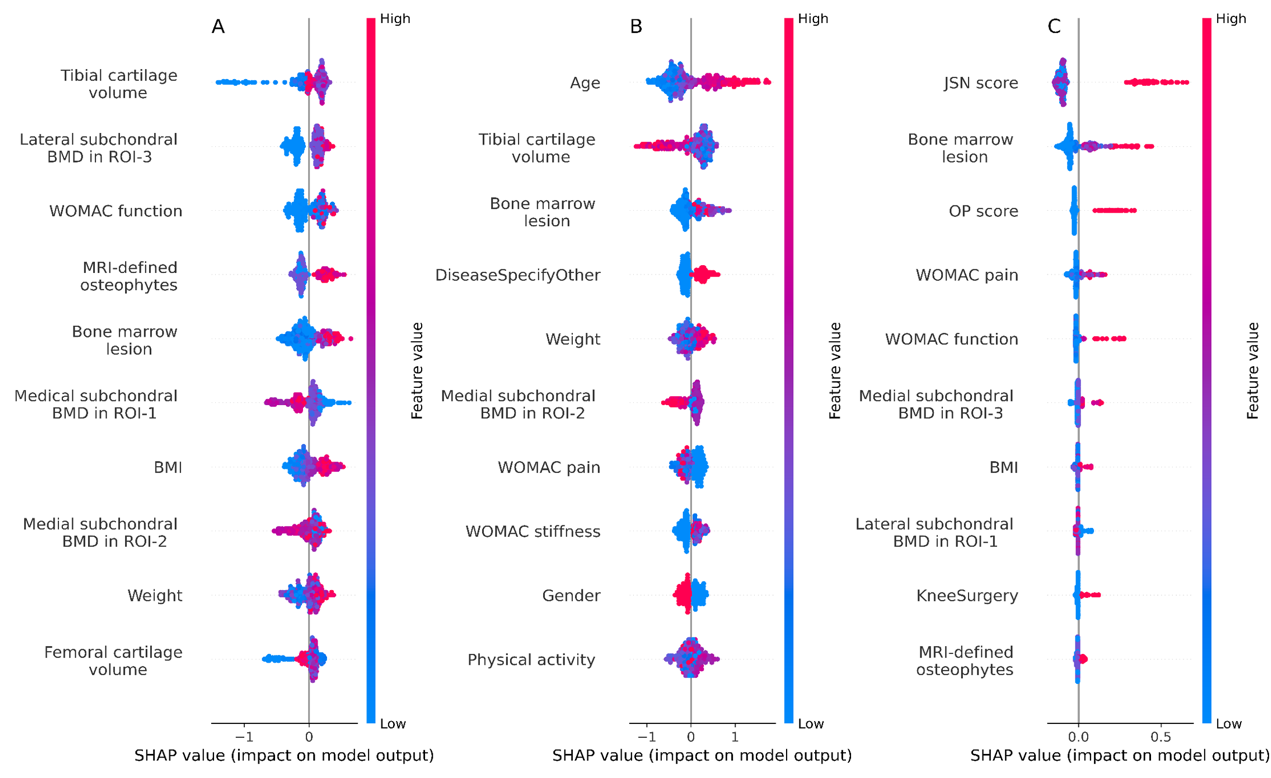


1. Body mass index [↑](#endnote-ref-1)
2. Western Ontario and McMaster Universities Osteoarthritis Index [↑](#endnote-ref-2)
3. Joint space narrowing [↑](#endnote-ref-3)
4. Region of interest [↑](#endnote-ref-4)
5. Magnetic Resonance Imaging [↑](#endnote-ref-5)
6. Western Ontario and McMaster Universities Osteoarthritis Index [↑](#endnote-ref-6)
7. Magnetic Resonance Imaging [↑](#endnote-ref-7)
8. Radiographic osteoarthritis [↑](#endnote-ref-8)
9. Osteoarthritis [↑](#endnote-ref-9)
10. Body mass index [↑](#endnote-ref-10)
11. Total knee replacement [↑](#endnote-ref-11)
12. Region of interest [↑](#endnote-ref-12)
13. 100*(tibial cartilage volume wave4- tibial cartilage volume wave1)/time of follow-up [↑](#endnote-ref-13)
14. Joint Space Narrowing [↑](#endnote-ref-14)
