# Supplementary material for: Predictive validity of consensus-based MRI definition of osteoarthritis plus radiographic osteoarthritis for the progression of knee osteoarthritis: A longitudinal cohort study
Source: Osteoarthr Cartil Open. 2025 Feb 15;7(2):100582. doi: 10.1016/j.ocarto.2025.100582 (PMC11889593; doi:10.1016/j.ocarto.2025.100582)
Supplement: Multimedia component 2 [file mmc2.docx]

**Supplementary text for:**

# Predictive validity of consensus-based MRI definition of osteoarthritis plus radiographic osteoarthritis for the progression of knee osteoarthritis: a longitudinal cohort study

Xing Xing^1 2^, Yining Wang^3^, Jianan Zhu^4^, Ziyuan Shen^1 3^, Flavia Cicuttini^5^, Graeme Jones^3^, Dawn Aitken^3^, Guoqi Cai^1 3^

1. *Department of Epidemiology and Biostatistics, School of Public Health, Anhui Medical University, Hefei 230032, Anhui, China*
2. *Department of Biostatistics, Johns Hopkins Bloomberg School of Public Health, Baltimore, MD, USA*
3. *Menzies Institute for Medical Research, University of Tasmania, Hobart 7000, Tasmania, Australia*
4. *Department of Biostatistics, School of Public Health, New York University, New York 10003, New York, USA*
5. *School of Public Health and Preventive Medicine, Monash University, Melbourne, Australia*

Correspondence to: Guoqi Cai, Department of Epidemiology and Biostatistics, School of Public Health, Anhui Medical University, Hefei 230032, Anhui, China, [Guoqi.Cai@utas.edu.au](mailto:Guoqi.Cai@utas.edu.au)

Dawn Aitken: [dawn.aitken@utas.edu.au](mailto:dawn.aitken@utas.edu.au); Graeme Jones: [graeme.jones@utas.edu.au](mailto:graeme.jones@utas.edu.au); Flavia Cicuttini: [Flavia.Cicuttini@monash.edu.au](mailto:Flavia.Cicuttini@monash.edu.au); Jianan Zhu: [jz4698@nyu.edu](mailto:jz4698@nyu.edu); Xing Xing: [kewinxing@outlook.com](mailto:kewinxing@outlook.com); Yining Wang: [yiining.wang@outlook.com](mailto:yiining.wang@outlook.com); Ziyuan Shen: [sshenzy@126.com](mailto:sshenzy@126.com).

**MRI measurements**

Magnetic resonance imaging-defined baseline osteophytes were evaluated by T2-weighted MRI for a previous study and were defined as focal bony excrescences on sagittal, axial, or coronal images, extending from a cortical surface. ^1^ This was conducted by an orthopedist with 5 years of experience. Osteophytes were graded as 0 (absent), 1 (<3mm), 2 (3-5mm), and 3 (>5mm) at the anterior, central weight-bearing and posterior margins of the femoral condyles and tibial plateaus at both medial and lateral sites, and the medial and lateral margins of the patella.^2^ A definite osteophyte was defined as grade 2 or higher at any site ^3^. Intraclass correlation coefficients (ICCs) for intra-observer reliability ranged from 0.94 to 0.97 and for inter-observer reliability from 0.90 to 0.96.^4^

Subchondral bone marrow lesions (BMLs) were assessed on T2-weighted MRI at baseline and defined as areas of hyperintensity in the subchondral bone in the medial tibial, medial femoral, lateral tibial, and lateral femoral sites ^5^. ICC for intra-observer reliability was 0.97 for this measure.^5^

Cartilage lesions at the medial tibial, medial femoral, lateral tibial, and lateral femoral sites were measured on T1-weighted MRI in the baseline. For each site, cartilage lesions were graded from 0 to 4 using the modified Outerbridge Score^6^ as follows: grade 0=normal cartilage; grade 1=focal blistering and intra cartilaginous low-signal-intensity area with an intact surface; grade 2=irregularities on the surface or bottom and loss of thickness <50%; grade 3=deep ulceration with loss of thickness >50%; grade 4=full-thickness chondral wear with exposure of subchondral bone. Any cartilage lesions from grade 1 to grade 3 were defined as partial thickness cartilage loss and grade 4 as full-thickness cartilage loss. Cartilage lesions were scored by a researcher with 5 years of experience in OA research. ICCs for intra-observer reliability ranged from 0.80 to 0.95.^7^

Meniscal lesions at baseline were assessed on T1-weighted MRIs as described previously.^8^ Meniscus tear and extrusion at any of the anterior, middle, and posterior horns were recorded. Meniscal lesions were measured by musculoskeletal radiologists from ArthroLab Inc (Montreal, Canada) ^9^. ICCs for intra- and inter-observer reliability ranged from 0.86 to 0.96 for meniscal tears and 0.85 to 0.92 for meniscal extrusions ^9^.

Effusion-synovitis was identified and assessed in STIR images. The observer (HGA) manually selected the magnetic resonance slice with the largest effusion-synovitis and determined the maximum cross-sectional area of the bright region by manually drawing contours around the outer edges^10^.

**Other factors**

Subchondral BMD (g/cm2) of the medial and lateral tibia for the right knee were measured using a Hologic Delphi densitometer (Waltham, MA, USA). Subchondral bone was defined as the entire tibia proximal to the head of the fibula. Based on our previous work, three regions of interest (ROIs) for both medial and lateral compartments were drawn manually to measure subchondral BMD using dual-energy X-ray absorptiometry (DXA) ^11^.

Smoking status (i.e. never, former, and current smoker) was defined based on two questions: ‘Have you ever been a ‘regular smoker’ (i.e. someone who has smoked at least 7 cigarettes, cigars, or pipes every week for at least 3 months)?’, and ‘Are you currently a ‘regular smoker’?’ ^12^.

Sociodemographic and lifestyle factors: Weight was measured using Seca Delta scales (Delta Model707; Seca, Hamburg, Germany) and height by Leicester stadiometer (Invicta, Leicester, UK.^13^ BMI was calculated (weight (kg)/height (m)2). Date of birth, sex, education level (highest attained), and employment and smoking status were assessed by questionnaire. Education was categorized into primary (no formal qualifications/ school or intermediate certificate), secondary (higher school or leaving certificate/trade/apprenticeship), and tertiary (certificate/diploma/ university degree/ higher university degree), and employment status into employed (employed or self-employed either full- or part-time), unemployed (home duties/student/ sole parent pension/ disability pension/unemployed), and retired.

To assess area-level socio-economic status, each participant’s residential address was matched to the corresponding Australian Bureau of Statistics (ABS) Census Collection District. ABS software (ABS, Canberra, Australia) was used to determine the Socio-Economic Indexes for Areas (SEIFA) value from the 2001 census for each participant.^14^ SEIFA is a collection of four separate indices, each constructed from different variables, which summarizes the characteristics of residents within an area (~250 households), thereby providing a single measure to rank the level of advantage and/or disadvantage at the area level, not of the individual level. For this study, we used the three SEIFA that are equivalized for both advantage and disadvantage: the Index of Relative Socioeconomic Advantage and Disadvantage (IRSAD), the Index of Education and Occupation (IEO), and the Economic Resources (IER).^14, 15^ The IRSAD is an aggregate of variables including, but not limited to, household income, car ownership, the number of one-parent families, and educational attainment. Similarly, the IEO includes the proportion of employed individuals within the area, educational attainment, and if employed, the type of occupation held. The IER measures area-based household income, markers of dwelling size, and car ownership^14, 15^. For each of IRSAD, IEO, and IER, quartile cut-points were based on the 2001 Tasmanian population.

The quality of life was assessed by a validated generic questionnaire, namely the Assessment of Quality of Life (AQoL-4D), was employed to assess the quality of life, of which the 12 items of four dimensions were transformed into a single utility score (0–1) via a multi-attribute utility instrument^16^. Comorbidities were collected by questionnaires^17^.

**References**

1. Zhu Z, Laslett LL, Jin X, Han W, Antony B, Wang X, et al. Association between MRI-detected osteophytes and changes in knee structures and pain in older adults: a cohort study. Osteoarthritis Cartilage 2017; 25: 1084-1092.

2. Peterfy CG, Guermazi A, Zaim S, Tirman PF, Miaux Y, White D, et al. Whole-Organ Magnetic Resonance Imaging Score (WORMS) of the knee in osteoarthritis. Osteoarthritis Cartilage 2004; 12: 177-190.

3. Schiphof D, Oei EH, Hofman A, Waarsing JH, Weinans H, Bierma-Zeinstra SM. Sensitivity and associations with pain and body weight of an MRI definition of knee osteoarthritis compared with radiographic Kellgren and Lawrence criteria: a population-based study in middle-aged females. Osteoarthritis Cartilage 2014; 22: 440-446.

4. Zhu Z, Ding C, Han W, Zheng S, Winzenberg T, Cicuttini F, et al. MRI-detected osteophytes of the knee: natural history and structural correlates of change. Arthritis Res Ther 2018; 20: 237.

5. Dore D, Quinn S, Ding C, Winzenberg T, Zhai G, Cicuttini F, et al. Natural history and clinical significance of MRI-detected bone marrow lesions at the knee: a prospective study in community dwelling older adults. Arthritis Res Ther 2010; 12: R223.

6. Recht MP, Kramer J, Marcelis S, Pathria MN, Trudell D, Haghighi P, et al. Abnormalities of articular cartilage in the knee: analysis of available MR techniques. Radiology 1993; 187: 473-478.

7. Dore DA, Winzenberg TM, Ding C, Otahal P, Pelletier JP, Martel-Pelletier J, et al. The association between objectively measured physical activity and knee structural change using MRI. Ann Rheum Dis 2013; 72: 1170-1175.

8. Berthiaume MJ, Raynauld JP, Martel-Pelletier J, Labonte F, Beaudoin G, Bloch DA, et al. Meniscal tear and extrusion are strongly associated with progression of symptomatic knee osteoarthritis as assessed by quantitative magnetic resonance imaging. Ann Rheum Dis 2005; 64: 556-563.

9. Raynauld JP, Martel-Pelletier J, Berthiaume MJ, Beaudoin G, Choquette D, Haraoui B, et al. Long term evaluation of disease progression through the quantitative magnetic resonance imaging of symptomatic knee osteoarthritis patients: correlation with clinical symptoms and radiographic changes. Arthritis Res Ther 2006; 8: R21.

10. Ahedi H, Aitken D, Blizzard L, Cicuttini F, Jones G. Quantification of hip effusion-synovitis and its cross-sectional and longitudinal associations with hip pain, MRI findings and early radiographic hip OA. BMC Musculoskelet Disord 2020; 21: 533.

11. Dore D, Ding C, Jones G. A pilot study of the reproducibility and validity of measuring knee subchondral bone density in the tibia. Osteoarthritis Cartilage 2008; 16: 1539-1544.

12. Shen Z, Wang Y, Xing X, Jones G, Cai G. Association of smoking with cartilage loss of knee osteoarthritis: data from two longitudinal cohorts. BMC Musculoskelet Disord 2023; 24: 812.

13. Cervo MM, Shivappa N, Hebert JR, Oddy WH, Winzenberg T, Balogun S, et al. Longitudinal associations between dietary inflammatory index and musculoskeletal health in community-dwelling older adults. Clin Nutr 2020; 39: 516-523.

14. Brennan SL, Winzenberg TM, Pasco JA, Wluka AE, Dobbins AG, Jones G. Social disadvantage, bone mineral density and vertebral wedge deformities in the Tasmanian Older Adult Cohort. Osteoporos Int 2013; 24: 1909-1916.

15. Nguyen HH, Wu F, Oddy WH, Wills K, Brennan-Olsen SL, Jones G, et al. Longitudinal associations of dietary patterns with sociodemographic and lifestyle factors in older adults: the TASOAC study. Eur J Clin Nutr 2021; 75: 759-767.

16. Wang Z, Jones G, Blizzard L, Aitken D, Zhou Z, Wang M, et al. Prevalence and correlates of the use of complementary and alternative medicines among older adults with joint pain. Int J Rheum Dis 2023; 26: 1760-1769.

17. Hoogeboom TJ, den Broeder AA, Swierstra BA, de Bie RA, van den Ende CH. Joint-pain comorbidity, health status, and medication use in hip and knee osteoarthritis: a cross-sectional study. Arthritis Care Res (Hoboken) 2012; 64: 54-58.
